# Synergistic action of peptidoglycan and teichoic acid synthesis inhibitors leads to cell death by oxidative damage

**DOI:** 10.1038/s42003-026-10124-z

**Published:** 2026-04-30

**Authors:** Yoshikazu Kawai, Yousef Dashti, Jeff Errington

**Affiliations:** 1 School of Medical Sciences, Faculty of Medicine and Health, University of Sydney, Sydney, NSW Australia; 2 Sydney Infectious Diseases Institute, University of Sydney, Sydney, NSW Australia

**Keywords:** Antibacterial drug resistance, Bacterial genetics

## Abstract

Peptidoglycan (PG) is the primary structural component of the bacterial cell wall. However, many bacteria can switch into a wall-deficient “L-form” state, whereby they grow without PG synthesis and become completely resistant to cell-wall-targeting antibiotics. Teichoic acids (TAs) are major glycopolymers of Gram-positive bacteria that are attached to either the PG wall (WTA) or the lipid membrane (LTA). We show that L-form growth does not require either WTA or LTA. However, it does require the TagO protein, which initiates the synthesis of WTA. Inhibiting TagO with the antibiotic tunicamycin triggers a metabolic shift resulting in oxidative damage-mediated cell death, and this is highly synergistic with perturbations of the PG synthetic system. UDP-GlcNAc, a key precursor for both PG and TA synthesis, controls a metabolic switch leading either to balanced growth or oxidative damage. Our results demonstrate the pivotal role of UDP-GlcNAc in the mechanism of killing by cell wall inhibition.

## Abstract (web summary)

Synergistic action of tunicamycin, an inhibitor of wall teichoic acid synthesis, in cell wall-free L-form bacteria sheds light on the role of cell wall precursors in triggering metabolic perturbations, oxidative damage and lethal toxicity.

## Introduction

Bacterial cells are surrounded by a cell wall, which protects cells from fluctuations in internal osmotic pressure and generates their characteristic shapes. In Gram-positive bacteria, the wall has two major components: (i) peptidoglycan (PG), whose biosynthetic pathway is a crucial target for our most effective antibiotics; and (ii) teichoic acids (TAs), which are anionic glycopolymers anchored to PG [wall teichoic acids (WTAs)] or the membrane [lipoteichoic acids (LTAs)].

PG is composed of glycan strands cross-linked by short peptides, forming a huge meshwork that covers the whole surface of the cell. Expansion of the cell during growth requires insertion of the disaccharide-peptide moiety of the PG precursor, lipid II. Lipid II is made in the cytoplasm via a series of highly conserved enzymes, then flipped to the outer surface of the membrane and inserted into the existing wall by the action of glycosyltransferase (GTase) and transpeptidase (TPase) enzymes^1^. The growth of PG also requires PG hydrolases, sometimes called autolysins, which break bonds in the PG meshwork, to enable cell expansion. In *Bacillus subtilis*, TAs are present in roughly equal amounts to PG^2^. The physical connection of TAs to PG and the cell membrane contributes to the final cell wall architecture, bringing about proper cell wall functionality. TagO initiates WTA biosynthesis by transferring GlcNAc from UDP-GlcNAc (which is also an important metabolite in lipid II synthesis) to an undecaprenyl phosphate (C_55_-P) carrier on the cytoplasmic face of the cell membrane to generate a GlcNAc-diphospholipid^3^. WTA polymer is then sequentially synthesised by a series of Tag enzymes, translocated across the membrane by the TagGH ABC transporter, and covalently attached to PG outside the cell^4^. Disruption of most of the *tag* genes is lethal^5,6^. However, *tagO*, which encodes the enzyme that catalyses the first step of the WTA biosynthetic pathway, is dispensable in *B. subtilis*^7^. Nevertheless, *tagO* mutants are severely compromised in cell growth, and they lose the ability to elongate, becoming rounded and swollen^8^. *tagO* mutation also suppresses the lethality of mutations in the later WTA genes^7^, although the basis of this suppression remains unclear. *Staphylococcus aureus* has a closely related WTA synthetic pathway, with a homologous series of genes and enzymes (e.g. *tarO* for *tagO*), but the polymer is built from ribitol phosphate, rather than glycerol phosphate^3^. The *tarO* gene is not essential, while the mutant cells show defects in septation and cell separation^9,10^. LTA is anchored to the cell membrane via a glycolipid, predominantly diglucosyl-diacylglycerol, which is produced from UDP-glucose and diacylglycerol. The LtaS protein or its minor paralogues polymerise the glycerol phosphate chain and link it to the glycolipid anchor. LTA synthesis is required for normal cell growth and division in both *B. subtilis* and *S. aureus*^11,12^.

Despite the critical importance of the wall for bacterial growth, many bacteria have the ability to switch to a wall-deficient state called the L-form^13^. Because they lack a PG wall, L-forms require isotonic conditions, but these are often met in human tissues and many other environments^14^. Importantly, L-forms are completely resistant to cell wall-active antibiotics, such as β-lactams, which prevent crosslinking of the glycan strands by binding to penicillin-binding proteins (PBPs), irreversibly inactivating their TPase activity^15^. L-forms have mainly been identified as antibiotic-resistant organisms in human samples and have been associated with a wide range of recurrent or persistent infectious diseases^14,16–18^. Importantly, L-forms are often able to switch back into the original walled state, providing a potential explanation for infection recurrence^19–22^. The molecular mechanisms underlying the generation and growth of L-forms have been studied in detail in recent years^23–36^.

*B. subtilis* has been used as a tractable model system for studying the molecular biology of L-forms. The transition from the original walled cells to the L-form occurs mainly through an event called “L-form escape”; the formation of a substantial cell wall lesion sufficient to allow the passage of a membrane-bound L-form cell (or protoplast) and its nuclear and cytoplasmic components, without serious damage to the chromosome or spillage of contents. Escape is dependent on the activity of autolytic enzymes or the presence of exogenous PG hydrolases, such as lysozyme^33,37,38^. Inhibition of PG synthesis by antibiotics (or mutations affecting PG synthesis) in emerging L-forms results in a metabolic diversion that stimulates the synthesis of membrane fatty acids^27,34^. This results in an increased rate of surface area relative to volume synthesis, which drives cell shape deformations and other dynamic membrane events leading to cell proliferation^27^. This unusual mode of division does not require the normally essential PG synthesis and cell division machinery^25,38,39^. However, growth is often prevented by oxidative damage (especially lipid peroxidation), because the perturbation of cellular metabolism upon PG inhibition also stimulates the generation of reactive oxygen species (ROS) as by-products of the central carbon oxidation pathway (i.e. glycolysis, TCA cycle and the respiratory chain (RC))^30,34^. Although oxidative damage is a critical impediment to L-form growth in many bacteria, this effect can be alleviated by reducing oxygen levels or iron availability, or by increasing gluconeogenic substrates in the growth environment^30,34,40^. We have also isolated several L-form-inducible strains in *B*. *subtilis* with mutations that downgrade or compensate for RC activity, such as *ispA* (involved in the synthesis of menaquinone, a central component of the RC), enabling L-forms to avoid the ROS-mediated lethality upon inhibition of PG synthesis^25,30^.

Although much is now known about the effects of PG perturbation on L-form physiology, it remained unclear whether TAs play any role in L-form growth or viability. In this work, we show that L-form growth does not require the assembly of WTA or LTA polymers, though TagO activity is required, despite being dispensable for growth in normal-walled cells. Growth deficiency by *tagO* inhibition in L-forms appears to depend on reduced utilisation of UDP-GlcNAc in cell wall synthesis, resulting in cell death mediated by oxidative damage. Our results demonstrate a pivotal role for the cell wall precursor UDP-GlcNAc in bacterial survival when cell envelope synthesis is perturbed.

## Results

### Tunicamycin inhibits the growth of L-forms

**Fig. 1:**
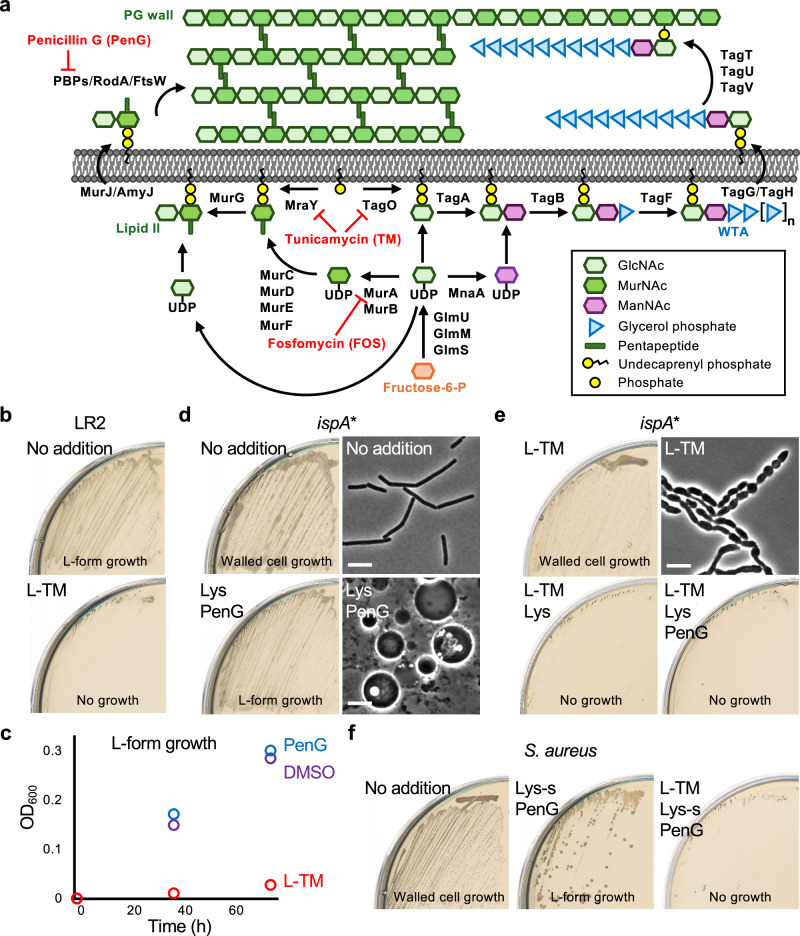
L-TM inhibits L-form growth. **a** Schematic representation of lipid II and WTA synthesis in *B. subtilis*. **b** L-form growth of *B*. *subtilis* strain LR2 (ispA^*^ *P*_*xyl*_-*murE*) was induced on osmo-protected NA/MSM plates (no added xylose), with or without 1 μg/ml TM (L-TM). The plates were incubated for 2–3 days at 30 °C. **c** Growth profiles of *B*. *subtilis* L-forms (LR2 strain) in an osmo-protected liquid NB/MSM medium containing 200 μg/ml PenG, 1 μg/ml TM (L-TM) or DMSO (control), as indicated. The cultures were incubated at 30 °C without shaking. OD_600_, optical density at 600 nm. Source data are provided as a Source Data file. **d** Growth of strain YK1395 (ispA^*^) on NA/MSM plates (No addition). L-forms were induced in the presence of 100 μg/ml lysozyme (Lys) and 100 μg/ml PenG. Phase contrast micrographs of YK1395 cells were obtained from cultures in liquid NB/MSM, with or without Lys and PenG. Scale bars represent 5 μm. **e** Growth of strain YK1395 (ispA^*^) on NA/MSM plates with L-TM (1 μg/ml TM), L-TM and Lys (100 μg/ml), or L-TM, Lys and PenG (100 μg/ml). A phase contrast micrograph of YK1395 cells was obtained from cultures in liquid NB/MSM with L-TM. Scale bar represents 5 μm. **f** *S*. *aureus* (RN4220) growth on NA/MSM plates. L-forms were induced in the presence of 2 μg/ml lysostaphin (Lys-s) and 100 μg/ml PenG, with or without L-TM (1 μg/ml), as indicated. The experimental data in this figure are representative of multiple independent experiments.

It was interesting to test whether *B. subtilis* L-forms have a requirement for WTA synthesis, since WTA is normally covalently attached to PG, which is usually not made in L-forms. In *B. subtilis*, a low concentration of the known cell wall inhibitor, tunicamycin (TM), specifically inhibits TagO protein, which is required for WTA synthesis^10,41^. At higher concentrations (>10 µg/ml), TM additionally blocks MraY, which is required for PG precursor synthesis (Fig. 1a)^42^. As shown in Supplementary Fig. 1a, growth of wild-type *B. subtilis* (168CA) on nutrient agar (NA) plates was severely impaired in the presence of low concentrations of TM (0.5–5.0 µg/ml), although cells were still viable, as evident from the presence of abundant, though small, colonies. However, colony formation was virtually eliminated at 10 µg/ml (Supplementary Fig. 1a). These results were consistent with the idea that the *tagO* gene is dispensable^7^, whereas *mraY* is essential^43^ and established that a low (e.g. 1 µg /ml) concentration of TM (“L-TM”) could be used to specifically inhibit TagO. In the equivalent liquid medium (nutrient broth, NB) with L-TM, the cells showed a bulging phenotype (Supplementary Fig. 1b), as expected for specific loss of *tagO* function^8,41^.

In previous work, we have established optimal conditions in which to culture *B. subtilis* in an L-form state. Strain LR2 has a xylose-inducible *P*_*xyl*_ promoter fused to the *murE* gene at its native locus, allowing lipid II synthesis to be shut down in the absence of xylose^27^. With xylose, the strain grows normally in the walled state (Supplementary Fig. 2a). LR2 also has a mutation in the *ispA* gene, which allows the strain to avoid ROS-mediated lethality upon inhibition of PG synthesis^30^. In the absence of xylose (lipid II synthesis OFF), this strain efficiently switches from walled to the L-form state when plated on NA supplemented with an osmo-protective solution, MSM (containing sucrose and Mg^2+^) (Fig. 1b and Supplementary Fig. 2a, b). However, we found that L-form growth was prevented on plates in the presence of L-TM (Fig. 1b). In liquid NB/MSM, L-form growth was completely resistant to the β-lactam penicillin G (PenG) (200 µg/ml), as expected, but it was largely inhibited in the presence of L-TM (Fig. 1c).

A *B. subtilis* strain carrying an *ispA* mutation can also efficiently switch into and maintain L-form growth in the presence of lysozyme and PenG (Fig. 1d, Lys/PenG)^33^. (Lysozyme promotes L-form escape by stripping the PG wall, while PenG, by blocking PG synthesis through inhibition of PBPs, prevents the resumption of PG synthesis). An *ispA* mutant strain grew well, albeit slowly, in the presence of L-TM on NA/MSM plates, and in NB/MSM, the walled cells showed a bulging phenotype (Fig. 1e, top panels). We also added lysozyme, or lysozyme and PenG with L-TM to stimulate the L-form switch, but no colonies appeared (Fig. 1e, bottom panels). Thus, L-TM appears to inhibit L-form growth, presumably by affecting TagO function.

WTA is also present in *S. aureus*, and TM inhibits TarO (equivalent to TagO) at low concentrations, and additionally MraY at higher concentrations^10^, just as for *B. subtilis*. We induced *S. aureus* L-forms by treating with lysostaphin (PG hydrolase active on *S. aureus*) and PenG on NA/MSM, as previously demonstrated^33^. The L-forms grew well on osmoprotective plates containing lysostaphin (Lys-s) and PenG, but no growth occurred in the presence of L-TM (Fig. 1f and Supplementary Fig. 2c).

### TagO is required for L-form growth but not via its role in WTA synthesis

**Fig. 2:**
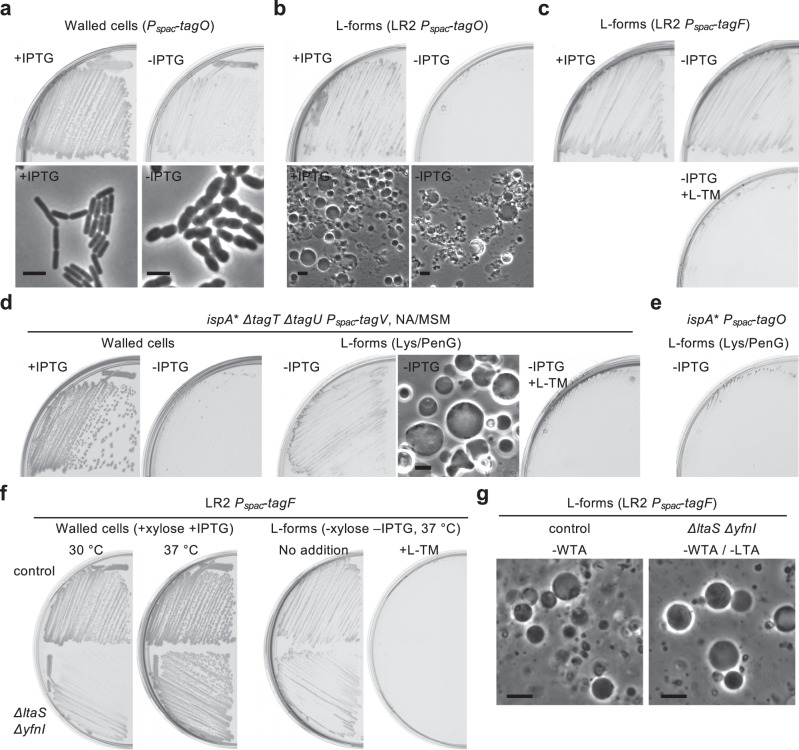
L-form growth requires TagO, but not WTA polymers. **a** Growth of strain YK1402 (*P*_*spac*_-*tagO*) on NA/MSM plates, with or without 1 mM IPTG. Phase contrast micrographs of YK1402 cells were obtained from cultures in liquid NB/MSM with or without IPTG. Scale bars represent 5 μm. **b** L-form growth of strain YK1359 (*P*_*spac*_-tagO ispA^*^ *P*_*xyl*_-*murE*) was induced on NA/MSM plates (no xylose), with or without 1 mM IPTG. Phase contrast micrographs were obtained from L-form cultures in liquid NB/MSM with or without IPTG. Scale bars represent 5 μm. **c** L-form growth of strain YK1361 (*P*_*spac*_-tagF ispA^*^ *P*_*xyl*_-*murE*) on NA/MSM plates (no xylose), with or without 1 mM IPTG, and in the presence of L-TM (1 μg/ml), as indicated. **d** Growth of strain YK2798 (*ΔtagT ΔtagU P*_*spac*_-tagV ispA^*^) on NA/MSM plates, with or without 1 mM IPTG. L-forms were induced in the presence of 100 μg/ml lysozyme (Lys) and 100 μg/ml PenG, with or without L-TM (1 μg/ml). A phase contrast image of L-form cells was obtained from the left plate adjacent to the micrograph. Scale bar represents 5 μm. **e** Inhibition of L-form growth of the strain YK1405 (*P*_*spac*_-tagO ispA^*^) on NA/MSM plates containing 100 μg/ml lysozyme (Lys) and 100 μg/ml PenG, in the absence of IPTG. **f** Growth of strains YK1361 (*P*_*spac*_-tagF ispA^*^ *P*_*xyl*_-*murE*) and YK2911 (Δ*ltaS* Δ*yfnI P*_*spac*_-tagF ispA^*^ *P*_*xyl*_-*murE*) on NA containing 0.5% xylose and 1 mM IPTG (walled cells), incubated at 30 or 37 °C. The L-form growth was induced on NA/MSM plates (no xylose) without IPTG, in the presence or absence of L-TM (1 μg/ml) at 37 °C. **g** Phase contrast micrographs of L-form cells were obtained from the plates shown in (**f**). Scale bars represent 5 μm. The experimental data in this figure are representative of multiple independent experiments.

As an alternative way to test the requirement for WTA on L-form growth in *B. subtilis*, we used a strain in which the natural promoter of the *tagO* gene was replaced with an IPTG-inducible *P*_*spac*_ promoter^44^. In the wild-type background (168CA), *tagO* repression in the absence of IPTG resulted in a slow growth phenotype on NA/MSM plates (Fig. 2a, top) and a bulging phenotype in liquid NB/MSM (Fig. 2a, bottom), as expected. However, when an LR2 background strain was switched into the L-form state (absence of xylose) on plates, growth was blocked by *tagO* repression (Fig. 2b, top), and in liquid NB/MSM, the L-forms showed abnormal aggregation and lysis (Fig. 2b, bottom). L-TM treatment generated similar morphological abnormalities in L-forms (Supplementary Fig. 2d).

After the action of TagO and TagA, TagB catalyses the transfer of a glycerol phosphate (GroP) unit from CDP-glycerol to ManNAc (Fig. 1a)^3^, which is derived from UDP-GlcNAc by the MnaA enzyme^8^. TagF then adds GroP units to the TagB product to assemble the polymer. These later-acting genes are essential for growth and rod-shaped morphology in normal-walled cells (Supplementary Fig. 3a, b)^6^, while the *tagO* gene is non-essential^7^. To test the essentiality of downstream *tag* genes in L-forms, we transferred *P*_*spac*_-*tagF* fusion into the LR2 strain background and tested the effects of repression on L-form growth. However, *tagF* repression had no significant effect (Fig. 2c, top), while L-TM treatment still inhibited L-form growth during *tagF* repression (Fig. 2c, bottom).

The LCP proteins (TagT, U and V in *B. subtilis*) carry out the final step of transferring WTA polymers from their lipid-linked precursor to PG, and a *tagTUV* triple mutant is lethal in normal-walled cells (Fig. 1a)^4^. The lethality upon inactivation of the *tagTUV* genes (using a strain carrying Δ*tagT* Δ*tagU P*_*spac*_*-tagV*) was also seen in an *ispA* mutant background (Fig. 2d). However, the triple mutant was able to grow in the L-form state in the presence of lysozyme and PenG, while this growth was prevented by L-TM (Fig. 2d, −IPTG + L-TM). We also confirmed that *tagO* repression impaired L-form growth induced by lysozyme and PenG (Fig. 2e).

Thus, loss of WTA does not necessarily prevent L-form growth: only loss of TagO function. It is striking that early vs. late blocks in WTA synthesis have opposite effects on the viability of walled cells and L-forms.

### Neither form of TA is required for L-form growth

We wondered whether LTA (a membrane-anchored TA of a similar polymeric structure to WTA) might also play a role in L-form growth (Supplementary Fig. 3c). In a glucose-rich medium, LtaS (the major LTA synthase) and YfnI (a stress-induced LTA synthase) elongate the LTA polymer^12,45,46^. We introduced Δ*ltaS* and Δ*yfnI* mutations into the LR2 strain with a *P*_*spac*_-*tagF* background. We then induced an L-form switch on NA/MSM plates at 37 °C. (We normally culture L-forms at 30 °C, but this strain exhibited a slow growth phenotype in the normal walled state at 30 °C (Fig. 2f).) Disruption of *ltaS* and *yfnI*, with *tagF* repression, resulted in only a slightly lower abundance or slower growth rates of L-forms (Fig. 2f, No addition), with no detectable morphological abnormalities (Fig. 2g). However, the growth was inhibited in the presence of L-TM (Fig. 2f, +L-TM). We also confirmed that deletion of *yqgS*, encoding the sporulation-specific minor LTA synthase^12^, has no significant effect on L-form growth (Supplementary Fig. 3d). It thus appears that the viability of L-forms is not dependent on assembled WTA or LTA polymers.

### L-TM sensitivity is increased by inhibition of the aPBP system but not the Rod system, in walled cells

**Fig. 3:**
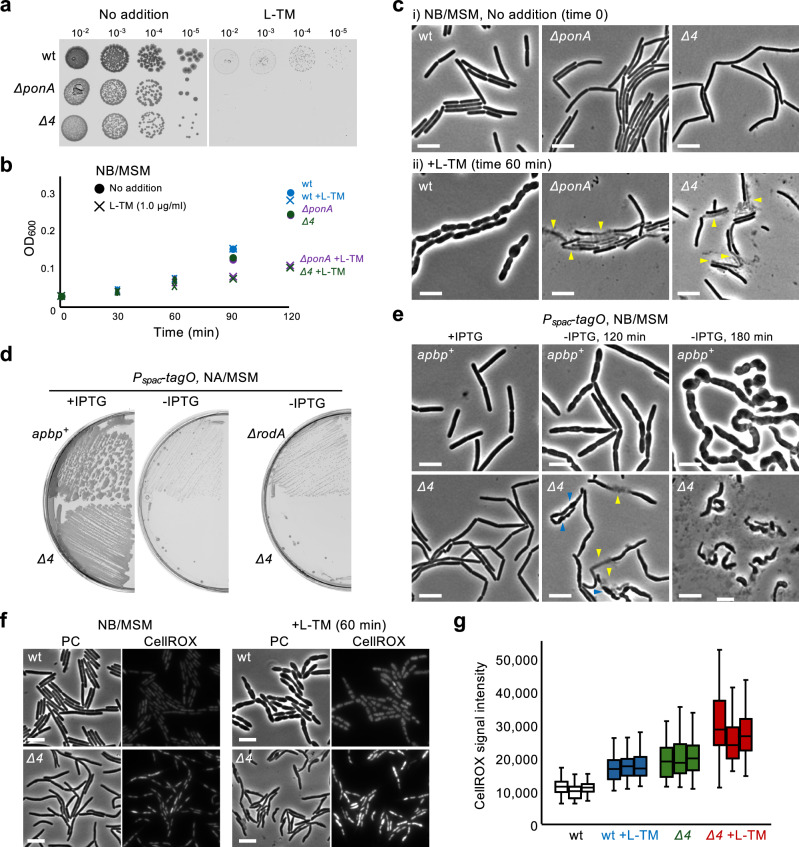
Increased ROS production by L-TM in a *Δ4* mutant. **a** L-TM sensitivity of the Δ*ponA* (YK1334) and Δ*4* (YK2239) mutants was compared to that of the wild type (168CA). Cell cultures of each strain were diluted in 10-fold series, and 6 μl spots were placed on NA plates, with or without L-TM (1 μg/ml). **b** Growth profiles of wild type (168CA), Δ*ponA* (YK1334) and Δ*4* (YK2239) in NB/MSM with or without L-TM (1 μg/ml), as indicated. OD_600_: optical density at 600 nm. Source data are provided as a Source Data file. **c** Phase contrast micrographs of *B. subtilis* strains were captured during the growth curve experiment shown in (**b**), at time 0 (i) and 60 min (ii) after the addition of L-TM, as indicated. The yellow arrowheads indicate a phase pale appearance. Scale bars represent 5 μm. **d** Growth of strains YK1402 (*P*_*spac*_-*tagO*), YK2584 (Δ*4 P*_*spac*_-*tagO*) and YK2837 (Δ*rodA P*_*spac*_-*tagO*) on NA/MSM plates with or without 1 mM IPTG, as indicated. **e** Phase contrast micrographs of strains YK1402 (*P*_*spac*_-*tagO*) and YK2584 (*Δ4 P*_*spac*_-*tagO*) were captured from cultures grown in NB/MSM with IPTG (+IPTG). These cultures were then diluted in fresh NB/MSM without IPTG and images were captured at 120 and 180 min, as indicated. The yellow arrowheads indicate a phase pale appearance, while the blue arrowheads indicate an abnormal swelling. Scale bars represent 5 μm. **f** ROS production in wild-type (168CA) and Δ*4* mutant (YK2239) cells. Wild-type and Δ*4* mutant cells were cultured in NB/MSM with or without L-TM (1 μg/ml) and then treated with CellROX Green treatment. Phase contrast (PC) and the corresponding CellROX fluorescent images were captured, as indicated. Scale bars represent 5 μm. **g** The signal intensity of green fluorescence in the cells shown in (**f**) was plotted as boxplots (*n* ≈ 100). The boxplots represent the upper and lower quartile values (boxes), the median (horizontal lines in the boxes) and the most extreme data points within 1.5 times the interquartile range (whiskers). Source data are provided as a Source Data file. The experimental data in this figure are representative of multiple independent experiments.

Previous studies have shown that TM treatment has a synergistic effect with β-lactam antibiotics (which prevent PG synthesis by inhibiting PBPs) on walled cells of *B. subtilis* and *S. aureus*, including methicillin-resistant *S. aureus* (MRSA)^10,47,48^. It is not completely clear why *B. subtilis* mutants affected in WTA synthesis take on a bulged or spherical shape in the walled state, although it has been suggested that they suffer from reduced availability of lipid II, which results in impairment of the MreB-RodA PG synthetic system required for cylindrical growth^49^. If so, the growth of such mutants should be dependent on the alternative aPBP PG synthetic system, which acts to insert new PG in a dispersed manner, leading to growth in a spherical form refs. ^50,51^. In *B. subtilis*, the four genes encoding aPBPs (*ponA*, *pbpD*, *pbpF* and *pbpG*) can be deleted (“*Δ4* mutant”) with only mild effects on cell growth and morphology^52^ (although growth is inhibited in the presence of a lower concentration of Mg^2+^ ^53^). As shown in Fig. 3a, both *ponA* (encoding the major aPBP) and Δ*4* mutant strains showed an increased sensitivity to L-TM on NA plates, as expected if L-TM impairs the functioning of the MreB-RodA system. The significant growth defects of both Δ*ponA* and Δ*4* mutants by L-TM were also seen in liquid medium, even in the presence of osmo-protective MSM (Fig. 3b). We then used phase contrast microscopy to examine the effects of L-TM on cell morphology (Fig. 3c). At 60 min after treatment with L-TM, wild-type cells showed bulging (Fig. 3c, ii). In contrast, although Δ*ponA* and Δ*4* mutant cells did not show significant shape changes, they exhibited extensive lysis (giving a phase pale effect; yellow arrowheads in Fig. 3c, ii), notably, even under osmo-protective conditions.

We then compared the effects of repressing *tagO* expression in mutants affected in the aPBP or Rod systems. As expected, Δ*4* mutant colonies did not emerge on NA/MSM plates when *tagO* was repressed. However, abundant colonies appeared in a Δ*rodA* background (Fig. 3d). In liquid NB/MSM, *apbp*^+^ cells (control) began to bulge within 120 min of *tagO* repression, but the cells continued to grow (Fig. 3e, top panels). In contrast, Δ*4* mutant cells showed a pale appearance (Fig. 3e, yellow arrowheads) and abnormal swelling effects (blue arrowheads) within 120 min after *tagO* repression. After 180 min, a few cells remained intact (phase dark), albeit with severe morphological defects, against a backdrop of completely lysed cells (Fig. 3e).

These observations suggest that culture growth after TagO inactivation is largely sustained by the aPBP system, supporting the idea that TagO (or WTA polymer) is required specifically for activity of the MreB-Rod system^54^. Furthermore, based on our previous work^40^, the development of phase pale cells, even under osmo-protective conditions, suggests that the Δ*4* mutant cells treated with L-TM are experiencing ROS-mediated cell death.

### Increased ROS production by L-TM in the absence of class A PBPs

We directly measured the effects of L-TM treatment on ROS levels using CellROX-green, a fluorescent dye for the detection of superoxide and hydroxyl radical^55–57^. Wild-type cells growing in NB/MSM showed a low intensity of CellROX fluorescence (Fig. 3f, g). After 60 min of L-TM treatment, the signal intensity had increased by about 1.6-fold, on average (Fig. 3f, g). In Δ*4* mutant cells, CellROX signals were higher than those of wild-type cells (approximately 1.8-fold) (Fig. 3f, g), consistent with cells suffering from oxidative stress when PG synthesis is perturbed^40^. However, signals were further increased upon L-TM treatment of the Δ*4* mutant (approximately 1.6-fold higher than untreated Δ*4*) (Fig. 3f, g), supporting the idea that the phase pale effect induced by *tagO* inhibition in Δ*4* mutant cells is mediated by an increase in ROS.

### TagO inhibition prevents L-form growth by stimulating ROS production

**Fig. 4:**
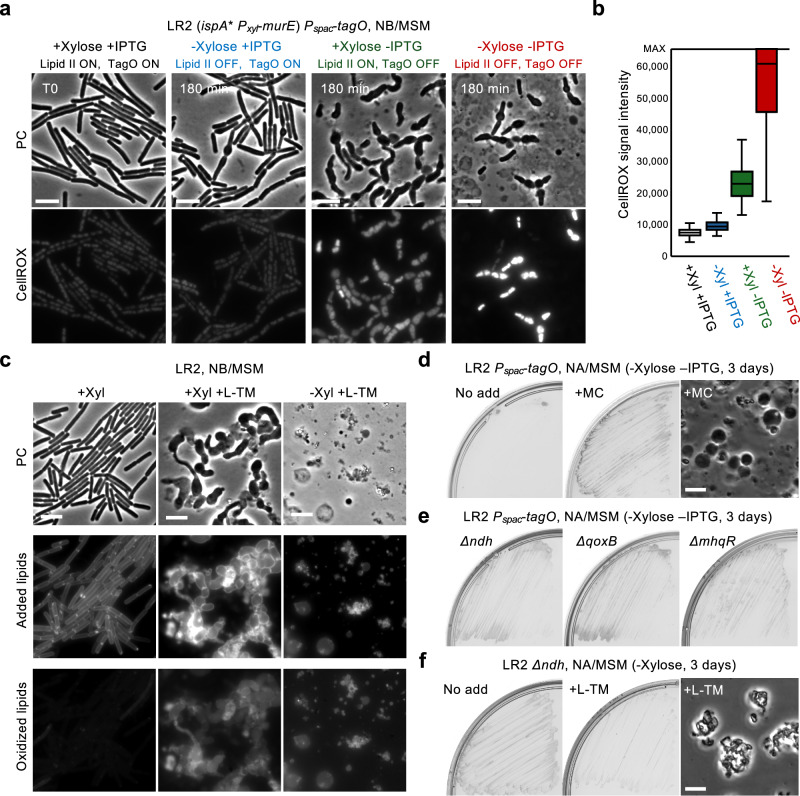
Increased ROS production by TagO inhibition during L-form switch. **a** Production of ROS with *tagO* repression during the L-form switch. YK1359 (*P*_*spac*_-tagO ispA^*^ *P*_*xyl*_-*murE*) was cultured in NB/MSM containing 0.5% xylose and 0.5 mM IPTG (+Xylose +IPTG). The culture was then diluted in fresh NB/MSM, either with or without xylose and IPTG. Phase contrast (PC) and the corresponding CellROX fluorescent images were then captured at 180 min, as indicated. Scale bars represent 5 μm. **b** The signal intensity of green fluorescence in the cells shown in (**a**) was plotted as boxplots (*n* ≈ 150). Boxplots represent the upper and lower quartile values (boxes), the median (horizontal lines in the boxes) and the most extreme data points within 1.5 times the interquartile range (whiskers). Source data are provided as a Source Data file. **c** Lipid peroxidation (LPO) during L-form switch. LR2 strain (ispA^*^ *P*_*xyl*_-*murE*) was cultured in NB/MSM with 0.5% xylose (+Xyl). The culture was diluted in fresh NB/MSM, either with or without xylose, in the presence of L-TM (1 μg/ml), incubated and then treated with C_11_-BODIPY^581/591^, after which images were taken. Scale bars represent 5 μm. **d** L-form growth during *tagO* repression in the presence of MC. YK1359 (*P*_*spac*_-tagO ispA^*^ *P*_*xyl*_-*murE*) was streaked on NA/MSM plates (with no xylose or IPTG) with or without MC (40 μg/ml), as indicated. The phase contrast micrograph of the L-form cells was obtained from the plate to the left of the micrograph. Scale bar represents 5 μm. **e** L-form growth during *tagO* repression in strains YK1464 (Δ*ndh P*_*spac*_-tagO ispA^*^ *P*_*xyl*_-*murE*), YK1465 (Δ*qoxB P*_*spac*_-tagO ispA^*^ *P*_*xyl*_-*murE*) and YK1526 (Δ*mhqR P*_*spac*_-tagO ispA^*^ *P*_*xyl*_-*murE*) on NA/MSM plates (with no xylose or IPTG), as indicated. **f** L-form growth in the presence of L-TM. YK1464 (Δ*ndh P*_*spac*_-tagO ispA^*^ *P*_*xyl*_-*murE*) was streaked on NA/MSM plates (no xylose) with or without L-TM (1 μg/ml). The phase contrast micrograph of L-form cells was obtained from the left plate adjacent to the micrograph. Scale bar represents 5 μm. The experimental data in this figure are representative of multiple independent experiments.

On the basis of the above results, it seemed that oxidative damage was responsible for the block in L-form growth resulting from TagO inhibition. To test this, we used a derivative of strain LR2 (*P*_*xyl*_-murE ispA*) carrying *P*_*spac*_-*tagO*. We cultured the strain in NB/MSM with xylose (lipid II synthesis ON) and IPTG (*tagO* expression ON). The cells showed only a weak CellROX fluorescence (Fig. 4a, b, +Xylose +IPTG). When the culture was diluted into the fresh NB/MSM containing IPTG but no xylose (*tagO* ON, lipid II OFF), the cells did not show a significant increase in the signals, but they started to bulge after 180 min (a typical early shape change during the L-form switch^33^) (Fig. 4a, b, -Xylose +IPTG). In the absence of IPTG but with xylose (*tagO* OFF, lipid II ON), the cells showed bulging, occasional lysis and an increase in the CellROX signals (Fig. 4a, b, +Xylose -IPTG). (The cell lysis upon *tagO* repression is probably due to insufficient lipid II synthesis in the LR2 background, see also below). However, in the absence of both IPTG and xylose (*tagO* OFF, lipid II OFF), many cells lysed, while in the intact (phase dark) cells, a dramatic increase in the CellROX signals was observed, with saturating signal intensity in about 40% of the cells (Fig. 4a, b, -Xylose -IPTG). Thus, TagO inhibition appears to substantially increase ROS production during the L-form switch, despite the normally protective effect of the *ispA* mutation^30^.

High levels of ROS can cause damaging lipid peroxidation (LPO), which alters the physiological properties of the cell membrane and generates a range of toxic effects in all cells, such as ferroptosis in eukaryotic cells^58^ or a phase pale effect in bacteria^40^. To test this, we took advantage of a fluorescent fatty acid analogue, C_11_-BODIPY^581/591^ ^59^. In a control experiment with the LR2 strain in the presence of xylose, no clear fluorescence of oxidised lipids was detectable in rods (Fig. 4c, +Xyl). In contrast, after L-TM treatment, cells of the LR2 strain began to bulge and lyse, and LPO became readily detectable (Fig. 4c, +Xyl +L-TM). After removal of xylose from the culture (but with continued L-TM), most of the cells lysed (Fig. 4c, -Xyl +L-TM). These results suggest that simultaneous inhibition of both lipid II synthesis and TagO leads to an enhanced degree of oxidative damage that overwhelms the normally protective effect of the *ispA* mutation.

We previously reported that an iron chelator, mirubactin C (MC), can lower intracellular iron levels^60^, leading to reduced oxidative damage and especially lipid peroxidation^40^. Strikingly, the deficiency of L-form growth upon *tagO* repression was significantly ameliorated in the presence of MC (Fig. 4d).

We then tested the effects of several previously isolated L-form promoting mutations that act by reducing ROS-mediated toxicity (i.e. *ndh*, *qoxB* and *mhqR*^30^), similar to that of the *ispA* mutation. *ndh* encodes a major NADH dehydrogenase, and *qoxB* encodes cytochrome *aa*_*3*_ quinol oxidase subunit I in the respiratory chain (RC) pathway. *mhqR* encodes a transcriptional repressor for genes induced by oxidative and/or electrophilic stress response^61^. As shown in Fig. 4e, the introduction of these mutations into the ispA* bearing LR2 strain rescued L-form growth during *tagO* repression. Thus, the lethal effects of *tagO* repression can be bypassed by combining ispA* with mutations further downregulating RC activity, upregulating oxidative stress response genes or chelating excess iron.

As shown in Fig. 4f, introducing an *ndh* mutation into the LR2 background partially rescued L-form growth in the presence of L-TM. However, growth was significantly reduced compared to the absence of L-TM (left panel), with many L-forms exhibiting abnormal aggregation. Treatment with L-TM may also affect L-form morphology in ways other than by inhibiting TagO or via the ROS-mediated pathway, at least under certain conditions.

**Fig. 5:**
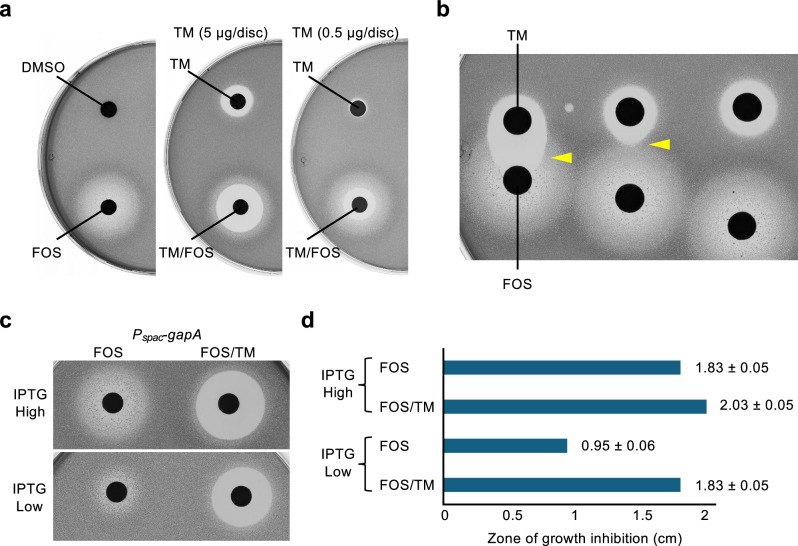
Synergistic combination of FOS and L-TM. **a** Disc diffusion assay of *B. subtilis* wild type (168CA) on NA plates using paper discs with 5 μl of DMSO (10%), 20 μg/μl FOS, 1 μg/μl TM and/or 0.1 μg/μl TM, as indicated. **b** Double-disc synergy test using paper discs with 5 μl of FOS (20 μg/μl) and TM (1 μg/μl), as indicated. The yellow arrowheads indicate the synergistic effect between FOS and TM. **c** Disc diffusion assay of YK1567 (*P*_*spac*_-*gapA*) on NA plates with 1 mM (High) or 0.01 mM IPTG (Low) using paper discs with 5 μl of FOS (20 μg/μl), and FOS and TM (1 μg/μl), as indicated. **d** Zones of growth inhibition (cm) were measured from four assays. The means and SD were shown. Source data are provided as a Source Data file. The experimental data in this figure are representative of multiple independent experiments.

Finally, we examined the effects of combining L-TM treatment with fosfomycin (FOS), an antibiotic that catalyses the transfer of phosphoenolpyruvate to UDP-GlcNAc in the first step of lipid II synthesis (Fig. 1a)^62^. As such, it blocks all PG synthesis, whether by class A PBPs or the MreB/Rod system. This antibiotic can also stimulate ROS-mediated toxicity in *S. aureus* and *B. subtilis*^63,64^. Based on the results above, we expected that FOS and L-TM should act synergistically, and indeed, as shown in Fig. 5a, the combination enhanced the killing effect over that of either single compound. The synergistic effects of FOS and TM were also confirmed by a double-disc synergy test (Fig. 5b, yellow alowheads). We previously showed that reducing glycolytic flux by partially repressing the *gapA* gene using *P*_*spac*_-*gapA* relieved the ROS toxicity due to impaired PG synthesis^34,40^. As shown in Fig. 5c, d, FOS treatment exhibited significantly reduced antimicrobial activity at a low IPTG concentration, as indicated by the smaller zone of growth inhibition around the FOS disc. Combining FOS with TM greatly enhanced the killing effect at both IPTG concentrations. Nevertheless, limiting glycolysis with low IPTG still conferred a level of resistance (Fig. 5c, d), consistent with the idea that both FOS and L-TM act to increase glycolytic flux, leading to endogenous ROS.

### L-TM treatment increases intracellular UDP-GlcNAc in L-forms

**Fig. 6:**
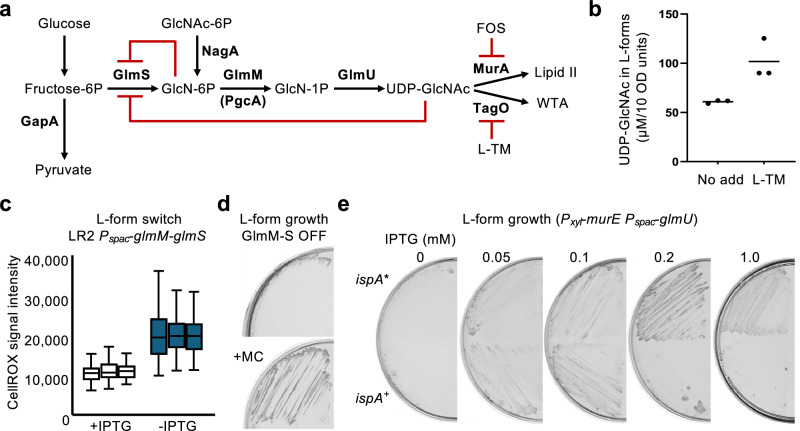
Critical role for UDP-GlcNAc in L-form viability. **a** A schematic representation of UDP-GlcNAc synthesis and its utilisation in lipid II and WTA synthesis in *B. subtilis*. UDP-GlcNAc is generated from fructose-6-phosphate (a glycolytic intermediate) through the action of GlmS, GlmM and GlmU enzymes. GapA is an essential enzyme in lower glycolysis. NagA catalyses the deacetylation of GlcNAc-6-phosphate to produce GlcN-6-phosphate. PgcA can act as a phosphoglucosamine mutase, catalysing the conversion of GlcN-6-phosphate to GlcN-1-phosphate^84^. UDP-GlcNAc inhibits GlmS activity^65,66^, while GlcN6P also inhibits the GlmS enzyme through a post-transcriptional feedback mechanism^85^. The cell wall inhibitors FOS and L-TM inhibit MurA and TagO, respectively. **b** The effect of L-TM on the concentration of intracellular UDP-GlcNAc in L-forms. L-forms (LR2 strain, ispA^*^ *P*_*xyl*_-*murE*) were cultured in NB/MSM. The L-form culture was then divided into two, with L-TM (5 μg/ml) added to one portion. Both cultures were incubated for 60 min, after which UDP-GlcNAc levels were measured. The means were obtained from three independent experiments. OD units = OD_600_ × culture volume. Source data are provided as a Source Data file. **c** Production of ROS with *glmM*-*glmS* repression during the L-form switch. YK1563 (*P*_*spac*_-*glmM*-glmS ispA^*^ *P*_*xyl*_-*murE*) were cultured in NB/MSM containing 0.5% xylose and 0.5 mM IPTG. This culture was diluted in fresh NB/MSM (no xylose), with or without IPTG, after which phase contrast (PC) and the corresponding CellROX-fluorescent images were captured at 180 min. The signal intensity of green fluorescence was plotted as boxplots (*n* ≈ 100). Boxplots represent the upper and lower quartile values (boxes), the median (horizontal lines in the boxes) and the most extreme data points within 1.5 times the interquartile ranges (whiskers). Source data are provided as a Source Data file. **d** L-form growth of the strain YK1563 (*P*_*spac*_-*glmM*-glmS ispA^*^ *P*_*xyl*_-*murE*) with *glmM*-*glmS* repression was induced on osmo-protected NA/MSM plates (no xylose and IPTG), in the presence or absence of MC (40 μg/ml). **e** The effect of *glmU* expression levels on L-form growth. YK2809 (*P*_*spac*_-*glmU P*_*xyl*_-*murE*) and YK2810 (*P*_*spac*_-glmU ispA^*^ *P*_*xyl*_-*murE*) were streaked on NA/MSM plates (no xylose) containing various concentrations of IPTG, as indicated. The plates were incubated for 3 days at 30 °C. The experimental data in this figure are representative of multiple independent experiments.

The nucleotide sugar UDP-GlcNAc, a key precursor for both lipid II and WTA synthesis, is generated from a glycolytic intermediate, fructose-6-phosphate, via the action of GlmS, GlmM and GlmU enzymes (Figs. 1a and 6a). Our previous work has suggested that reduced UDP-GlcNAc utilisation for PG synthesis contributes to ROS toxicity^40^, presumably because accumulation of UDP-GlcNAc results in downregulation of GlmS activity (Fig. 6a)^65,66^ and thus increased flux through glycolysis. As TagO also consumes UDP-GlcNAc in initiating WTA synthesis (Figs. 1a and 6a), inhibiting TagO could also result in increased UDP-GlcNAc accumulation, particularly in cells in which its utilisation for the PG pathway is blocked. We therefore measured the intracellular levels of UDP-GlcNAc in growing L-forms using the LR2 strain. After 60 min of L-TM treatment, the intracellular UDP-GlcNAc levels were significantly higher than in L-forms cultured without L-TM treatment (Fig. 6b), consistent with the idea that increased UDP-GlcNAc concentration contributes to the toxic effect of TagO inhibition in L-forms.

To explore the link between increased UDP-GlcNAc and ROS further, we directly limited expression of the *glmM-glmS* (*glmM*-*S*) operon using a previously described *P*_*spac*_*-glmM-S* construct^34^. We cultured a derivative of strain LR2 (*P*_*xyl*_-murE ispA*), also containing the *P*_*spac*_-*glmM*-*S* construct, in NB/MSM with xylose and IPTG. When the culture was diluted into fresh NB/MSM containing IPTG (*glmM* operon ON) but no xylose, the cells showed a weak CellROX fluorescence (Fig. 6c +IPTG), while in the absence of IPTG (*glmM* operon OFF), intracellular ROS levels were significantly increased. Noteably, the deficiency of L-form growth following *glmM*-*glmS* repression was ameliorated in the presence of MC (Fig. 6d), supporting the idea that the block in L-form growth following inhibition of the UDP-GlcNAc pathway is due to ROS-mediated toxicity.

### UDP-GlcNAc as a key factor in L-form viability

We previously showed that downregulation of *glmU* (via a *P*_*spac*_-*glmU* construct), encoding the key final enzyme required for synthesis of UDP-GlcNAc (Figs. 1a and 6a), could bypass the lethal ROS toxicity resulting from impaired PG synthesis (FOS treatment or mutation of the *mbl* [*mreB*-like] gene)^40^. To test whether *glmU* repression could also support the growth of L-forms, we introduced the *P*_*spac*_-*glmU* construct into *B*. *subtilis* strains carrying *P*_*xyl*_-*murE*, with or without an *ispA* mutation. We then titrated the levels of IPTG required for L-form growth on NA/MSM plates in the absence of xylose (lipid II synthesis OFF). As shown in Fig. 6e, no growth occurred when *glmU* expression was completely blocked (0 IPTG), regardless of whether an *ispA* mutation was present, presumably because UDP-GlcNAc is a critical sugar donor for various cellular pathways^67^. At high IPTG concentrations (above 0.2 mM), L-form growth occurred only in the strain with the *ispA* mutation. However, at lower IPTG concentrations (0.05 and 0.1 mM), L-form growth occurred even in the *ispA*^+^ cells (Fig. 6e), consistent with the idea that UDP-GlcNAc levels play a critical role in the metabolic shift leading to ROS toxicity during L-form growth.

## Discussion

Previous studies have shown that the first enzyme of the WTA synthesis pathway, TagO, is dispensable for the growth of normal-walled *B*. *subtilis* and *S*. *aureus*^7,9^. In contrast, the later-acting proteins responsible for polymer formation and export are essential for growth. However, these late genes become dispensable in a *tagO* (or *tarO* in *S*. *aureus*) null background^7,9^, suggesting either an accumulation of toxic intermediates or sequestration of a shared precursor, such as undecaprenol phosphate, which is also used in lipid II synthesis. The detailed mechanism remains to be established. Nevertheless, it seems that WTAs are not essential for the walled growth of *B. subtilis* or *S. aureus*. The situation for LTA is clearer, and again, these molecules are not essential at least under certain conditions^12,68^. Nevertheless, we previously showed that loss of both WTA and LTA is lethal in *B. subtilis*^12^.

Surprisingly, we have now found that targeting the first step of WTA synthesis, either by L-TM treatment or repression of *tagO*, is lethal in L-forms, whereas a late block is not. However, the *tagO* lethality turns out to be due to ROS-mediated toxicity and can be suppressed in various ways, based on results we have described previously for various cell wall targeting factors^30,34,40^. Importantly, we have now established that L-form proliferation does not require either of the anionic polymer forms, WTA or LTA.

Previous studies have shown that L-TM treatment has a synergistic effect with β-lactam antibiotics^10,48^. We can now clarify that the synergy specifically arises from inhibition of aPBP activity, rather than MreB/Rod activity. Thus, L-TM treatment or *tagO* depletion led to growth arrest in an aPBP mutant strain (Δ*4*) but not in a *ΔrodA* mutant. Presumably, loss of WTA, which generally results in a round phenotype, somehow results in inhibition of the MreB/Rod system^49^. aPBP activity then synthesises PG in an unguided pattern, leading to spherical cells. We previously showed that the aPBP system appears to be more resilient than the MreB/Rod system to a reduction in PG precursor synthesis^38^. So, a reduction in the availability of precursors could contribute to this effect. Alternatively, Barber et al.^54^ have recently suggested that loss of WTA directly impacts MreB/Rod activity while stimulating aPBP (PonA) activity.

Ultimately, many of the results we described suggest that L-TM or *tagO* depletion results in an increase in ROS, and that this can act synergistically with the ROS generated by perturbation of PG synthesis, as we have described in detail previously^30,34,40^. Thus, the combination of L-TM and inactivation of aPBPs led not only to growth arrest, due to loss of all PG synthesis, but also to a large increase in ROS. Similar results were obtained from a combination of FOS and L-TM. This synergy can also explain our initially surprising finding of the sensitivity of L-forms to L-TM. We suggest that changes in the cellular levels of UDP-GlcNAc play a critical role in triggering ROS toxicity due to perturbations of cell envelope synthesis.

**Fig. 7:**
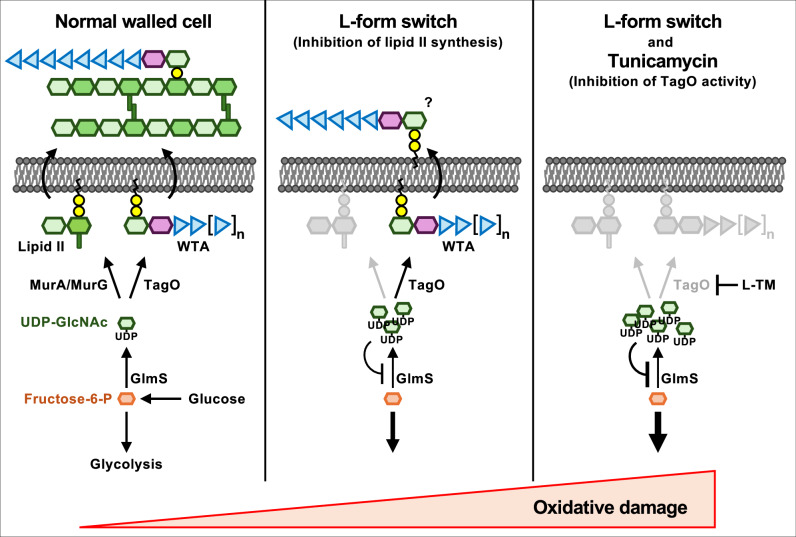
TagO inhibition enhances oxidative damage in L-forms. A schematic representation of the increased oxidative damage and its critical connection with the intracellular level of UDP-GlcNAc during the L-form switch. GlmS controls the entry of the glycolytic metabolite fructose-6-phosphate into the UDP-GlcNAc biosynthesis pathway. Increasing UDP-GlcNAc levels, by reducing its utilisation in cell wall synthesis (Lipid II and or WTA, with synergy), acts as a feedback inhibitor on GlmS activity. This leads to an increased level of oxidative damage through the catabolic burst, thereby preventing L-form growth.

Most treatments that result in the generation of L-forms of *B. subtilis*, and it seems generally in bacteria, involve the inhibition of PG synthesis or assembly^22,23,25,28,29,32,33,69^. We have shown previously that the inhibition of PG synthesis in L-forms increases carbon flux into lower glycolysis, resulting in oxidative stress via electron leakage from the RC^30,34^. The consequent oxidative damage generally contributes largely to the toxicity of cell wall inhibitors^40,64,70–75^. Our present results provide a possible mechanism for this effect. In Gram-positive bacteria, UDP-GlcNAc is used for building two or more important cell envelope structures, including the multi-layered PG (>20% of the total cell dry weight^76^) and cell surface glycopolymers (e.g. TAs and capsular polysaccharides). We have shown that the growth of L-form cells is dependent on the ability of the UDP-GlcNAc biosynthesis pathway to consume sufficient amounts of fructose-6-phosphate to prevent the catabolic burst leading to ROS production (Fig. 7). Entry of the glycolytic metabolite fructose-6-phosphate into the UDP-GlcNAc pathway is controlled by GlmS^67,77^. However, the downstream metabolite, UDP-GlcNAc, can inhibit this process by preventing GlmS activity, through the action of highly conserved GlmR/YvcJ proteins^65,66^. This regulatory structure provides an important mechanism for regulating the synthesis of PG and WTA, and ensuring an appropriate balance of sugar phosphate intermediates with other cellular needs. In line with the crucial role of metabolic direction and its flux in L-form survival, repressing the expression of the *glmS* (*glmM*-*glmS* operon) resulted in ROS-mediated growth inhibition (Fig. 6c, d). Increasing UDP-GlcNAc levels, by reducing its utilisation in cell wall synthesis (PG and or WTA, which act synergistically), also resulted in oxidative damage (Figs. 4 and 5). As cell wall synthesis is a major drain on cellular resources, it is not surprising that preventing fructose-6-phosphate from entering the cell wall pathway results in dramatic changes to cell physiology. We do not exclude the possibility that UDP-GlcNAc is also consumed in other pathways. Nevertheless, L-form growth is enabled by uncoupling the toxic effect associated with metabolic perturbations triggered by changes in UDP-GlcNAc levels during cell wall inhibition.

Interestingly, in Gram-negative diderm bacteria such as *Escherichia coli*, the function of the outer membrane (OM), which consists of outer membrane proteins and lipopolysaccharide (LPS), appears to be crucial for L-form survival and growth^21,69,78^. The biosynthesis of LPS begins with the acylation of the shared precursor UDP-GlcNAc to form lipid A^79^, and UDP-GlcNAc also serves as a precursor for assembly of the enterobacterial common antigen^80^, which is anchored in the outer leaflet. Thus, an increase in intracellular UDP-GlcNAc levels may also contribute to the growth deficiency in Gram-negative L-forms when OM synthesis is perturbed. In *E*. *coli*, UDP-GlcNAc does not appear to directly inhibit GlmS; instead, glucosamine-6-phosphate (the product of GlmS) acts a potent inhibitor of GlmS synthesis^81,82^. Nevertheless, it will be interesting in the future to explore the relationship between the essentiality of OM biogenesis for L-form growth and UDP-GlcNAc utilisation in diderm bacteria.

The results described above shed new light on the mechanism of killing by tunicamycin and extend our general understanding of how cell-wall-active antibiotics kill bacterial cells. Despite their variety of specific targets, including cytoplasmic enzymes for precursor synthesis and extracellular factors required for cell wall assembly, killing appears to occur by a common mechanism involving metabolic dysfunction leading to oxidative damage. This has important implications for understanding not only mechanisms of resistance and tolerance but also the development of new agents working on wall synthesis, or “adjuvants” that might enhance the oxidative damage. Also, we show that combinations of cell-wall-targeting antibiotics (e.g. FOS and L-TM) can act synergistically via increased production of ROS. Finally, our results also demonstrate, generally, the power of using L-form bacteria, which can grow in the complete absence of cell wall synthesis, to investigate cellular mechanisms and antibiotic mode of action.

## Methods

### Bacterial strains and growth conditions

The bacterial strains in this study are listed in Supplementary Table 1. DNA manipulations and transformations were carried out using standard methods. Nutrient agar and broth (NA and NB; Oxoid) were used for bacterial growth at 30 or 37 °C. L-form growth was induced on isotonic NA plates or NB medium containing MSM at 30 °C without shaking. NA/MSM and NB/MSM were prepared by mixing equal volumes of 2x NA (or 2x NB) and 2x MSM (40 mM magnesium chloride, 1.0 M sucrose, and 40 mM maleic acid, pH 7.0). 1 µg/ml FtsZ inhibitor, 8J^83^, was added to L-form medium to prevent the growth of walled cells when required. For selections of *B*. *subtilis* mutants, antibiotics were added to media at the following concentrations: 1.5 µg/ml erythromycin, 5 µg/ml chloramphenicol, 60 µg/ml spectinomycin or 2.5 µg/ml kanamycin. The concentration of kanamycin was increased to 10 µg/ml in the presence of added Mg^2+^ or MSM (containing 20 mM Mg^2+^). IPTG and xylose were supplemented, as appropriate. MC used in this study were purified or synthesised previously^60^.

### Disk diffusion assay

*B. subtilis* cells were grown in NB medium with appropriate requirements to an OD_600_ of 0.6–0.8. 100 μl of the culture (~1.0 × 10^9^ CFU/ml) was mixed with 50 ml of molten NA containing the necessary requirements, after which the cells and agar were poured onto the plates. Whatman Antibiotics Assay Discs (6 mm diameter) were used for the assays.

### Detection of ROS

ROS was detected using a CellROX Green (Thermo Fisher Scientific)^40^. *Bacillus subtilis* strains were cultured in NB/MSM (or NB) with appropriate requirements. To detect ROS (superoxide and hydroxyl radical), 1 ml of the cultures was incubated with 5 μM CellROX Green for 30 min at 37 °C. The cells were harvested by centrifugation and washed three times with fresh NB/MSM (or NB) before being used for microscopic analysis. CellROX Green is a proprietary oxidation-sensitive dye whose fluorescence quantum yield at 500–550 nm after excitation at 488 nm increases dramatically on oxidation in the presence of dsDNA^55^.

### Detection of Lipid peroxidation

Lipid peroxidation was detected using a fluorescent probe C_11_-BODIPY^581/591^ (Thermo Fisher Scientific)^30^. *B. subtilis* strains were cultured in NB/MSM. To detect Lipid peroxidation, 1 ml of the cultures was incubated with 5 μM C_11_-BODIPY^581/591^ for 60 min. The cells were harvested by centrifugation and washed three times with fresh NB/MSM before being used for microscopic analysis.

### Microscopy and image analysis

For live cell snapshot imaging, bacterial cells were mounted on microscope slides covered with a thin film of 1.2% agarose in water, or in NB/MSM. All microscopy experiments were performed on a Nikon Inverted Research Microscope ECLIPSE Ti2 equipped with a Nikon CFI Plan Apochromat DM Lambda 100× oil objective and a Teledyne Photometrics Prime BSI camera, using Nikon NIS-Elements AR software, as previously described^64^. Images were analysed and processed using FIJI (version 2.9.0/1.53t)

### Measurement of intracellular UDP-GlcNAc levels

*B. subtilis* L-forms were cultured in NB/MSM containing 100 μg/ml PenG until the optical density at 600 nm (OD_600_) reached 0.15. The L-form culture was then divided into two portions, with L-TM (5 μg/ml) added to one of them. Both cultures were then incubated for 60 min. Cells from 30 ml of each culture were harvested by centrifugation. The pellet was resuspended in 1 ml of fresh NB/MSM, washed, and then resuspended in 75 μl of 5% trichloroacetic acid. The suspension was incubated at room temperature for 20 min with shaking at 500 rpm. The mixture was centrifuged for 10 min at 15,871 × *g*, and the resulting supernatant was neutralised with the addition of 11.25 μl of a KOH (2.5 M)/K_2_HPO_4_ (1.5 M) solution for LC-MS analysis, as previously described^40^. UDP-GlcNAc concentrations were determined using Agilent MassHunter Quantitative Analysis 10.2 software based on a standard curve generated by analysing known concentrations of UDP-GlcNAc (Sigma-Aldrich) under identical LC-MS conditions.

A Thermo Scientific TSQ Altis Triple Quadrupole Mass Spectrometer equipped with an Agilent Poroshell 120 HILIC-Z (2.1 × 100 mm,1.9 µm) column was used for UDP-GlcNAc analysis. Mobile phases consisted of acetonitrile (A) and 0.1% formic acid in water (B). The flow rate was set at 0.2 mL/min. The mobile phase gradient was held at 10% A/90% B for 2 min, then decreased to 10% B over 18 min, followed by a rapid return to 90% B within 1 min. The column was then equilibrated for 9 min before the next run. MS acquisition was performed in negative ion mode. The dwell time was 100 ms, with the minimum chromatographic peak width set to 30 s. The ion transfer tube and vaporiser temperatures were set to 325 °C and 275 °C, respectively. The source voltage was −3500 V, and the RF lens voltage was optimised to 115 V. The centre mass (m/z) for the SIM precursor was optimised at 605.929.

### Statistics and reproducibility

The images were edited and analysed using the Fiji software. The fluorescent intensity was measured from 100 ~ 50 cells at a single time point using Fiji. For population-level assays (growth and disk diffusion assays), each experiment was conducted with at least two biological replicates and independently repeated at least twice. No statistical methods were used to predetermine the sample size, and the experiments were not randomised.

### Reporting summary

Further information on research design is available in the Nature Portfolio Reporting Summary linked to this article.

## Supplementary information

Supplemental Material

Description of Additional Supplementary Files

Supplementary Data

Reporting Summary

Transparent Peer Review File

## Data Availability

All data generated in this study are provided in the Supplementary Information/Source Data file. The numerical source data for the figures can be found in the Supplementary Data.
