# Supplementary material for: Synergistic action of peptidoglycan and teichoic acid synthesis inhibitors leads to cell death by oxidative damage
Source: Commun Biol. 2026 Apr 30;9:912. doi: 10.1038/s42003-026-10124-z (PMC13338364; doi:10.1038/s42003-026-10124-z)
Supplement: Supplementary file 1 — Supplemental Material [file 42003_2026_10124_MOESM1_ESM.pdf]

Supplementary Information for

Synergistic action of peptidoglycan and teichoic acid synthesis inhibitors leads to cell death by oxidative damage

Yoshikazu Kawai[1,2]*, Yousef Dashti[1,2], Jeff Errington[1,2]*

[1]Faculty of Medicine and Health, University of Sydney, Sydney, NSW, 2015, Australia

[2]Sydney Infectious Diseases Institute, University of Sydney, Sydney, NSW, 2015, Australia

*Corresponding authors

yoshikazu.kawai@sydney.edu.au

jeffery.errington@sydney.edu.au

Contents:

Supplementary Figs. 1-3, Supplementary Table 1 and Supplementary References.

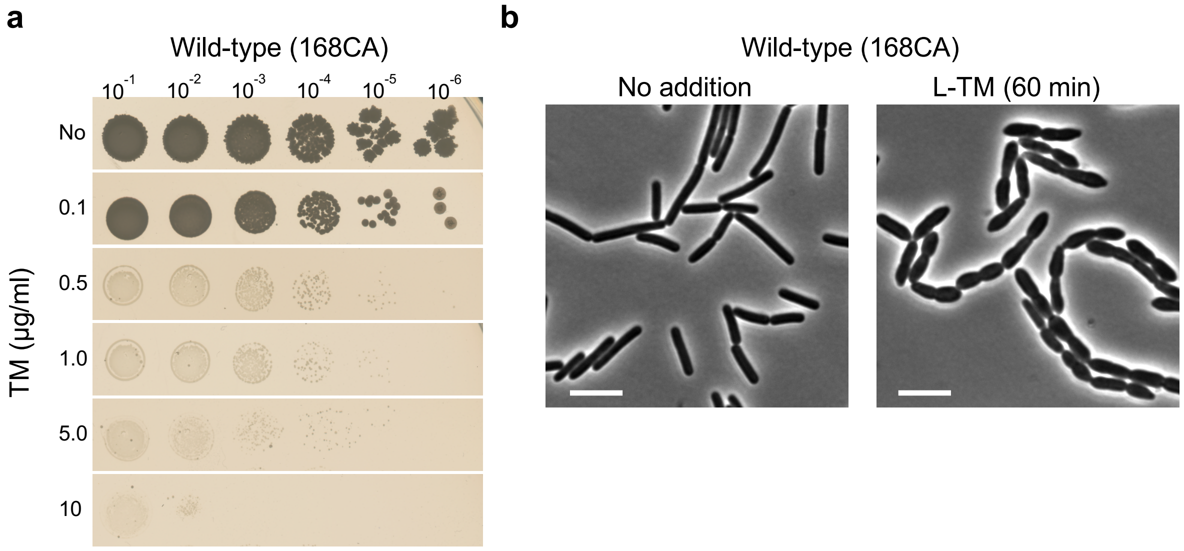

Supplementary Figure 1. The effects of tunicamycin on the growth and cell morphology of *B. subtilis*

a. TM sensitivity of *B. subtilis* wild type (168CA). A cell culture of 168CA was diluted in 10-fold series and 6 μl spots were placed on NA plates containing various concentrations of TM.

b. Phase contrast micrographs of 168CA in NB with or without L-TM (1 μg/ml).

The experimental data in this figure are representative of multiple independent experiments.

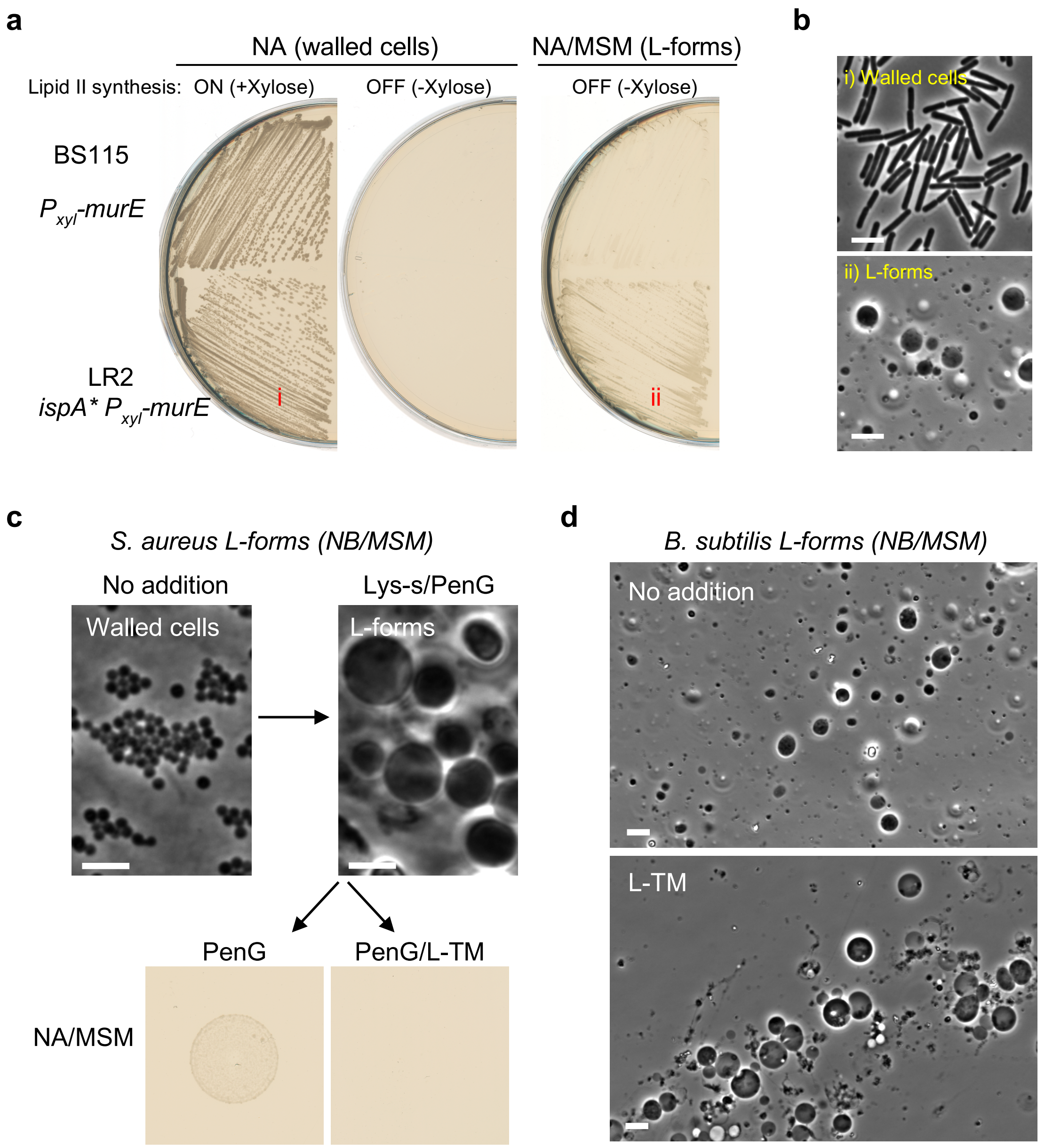

Supplementary Figure 2. L-form growth in *B. subtilis* and *S. aureus*

a. L-form growth is induced in the presence of an *ispA* mutation under osmo-protected conditions. BS115 (P_{xyl}-*murE*) and LR2 (*ispA** P_{xyl}-*murE*) were streaked on NA or NA/MSM, either with or without xylose, as indicated.

b. Phase contrast micrographs of *B. subtilis* cells were obtained from the plates of panel a (i and ii), as indicated. Scale bar represents 5 µm.

c. *S. aureus* (RN4220) L-forms were generated in NB/MSM by treating with 2 µg/ml lysostaphin (Lys-s) and 100 µg/ml PenG. 10 µl spots of the L-form culture were placed on NA/MSM plates containing PenG, in the presence or absence of L-TM (1 µg/ml).

d. Phase contrast micrographs of *B. subtilis* L-form cells (LR2) in liquid NB/MSM, in the presence or absence of L-TM (1 µg/ml). Scale bar represents 5 µm.

The experimental data in this figure are representative of multiple independent experiments.

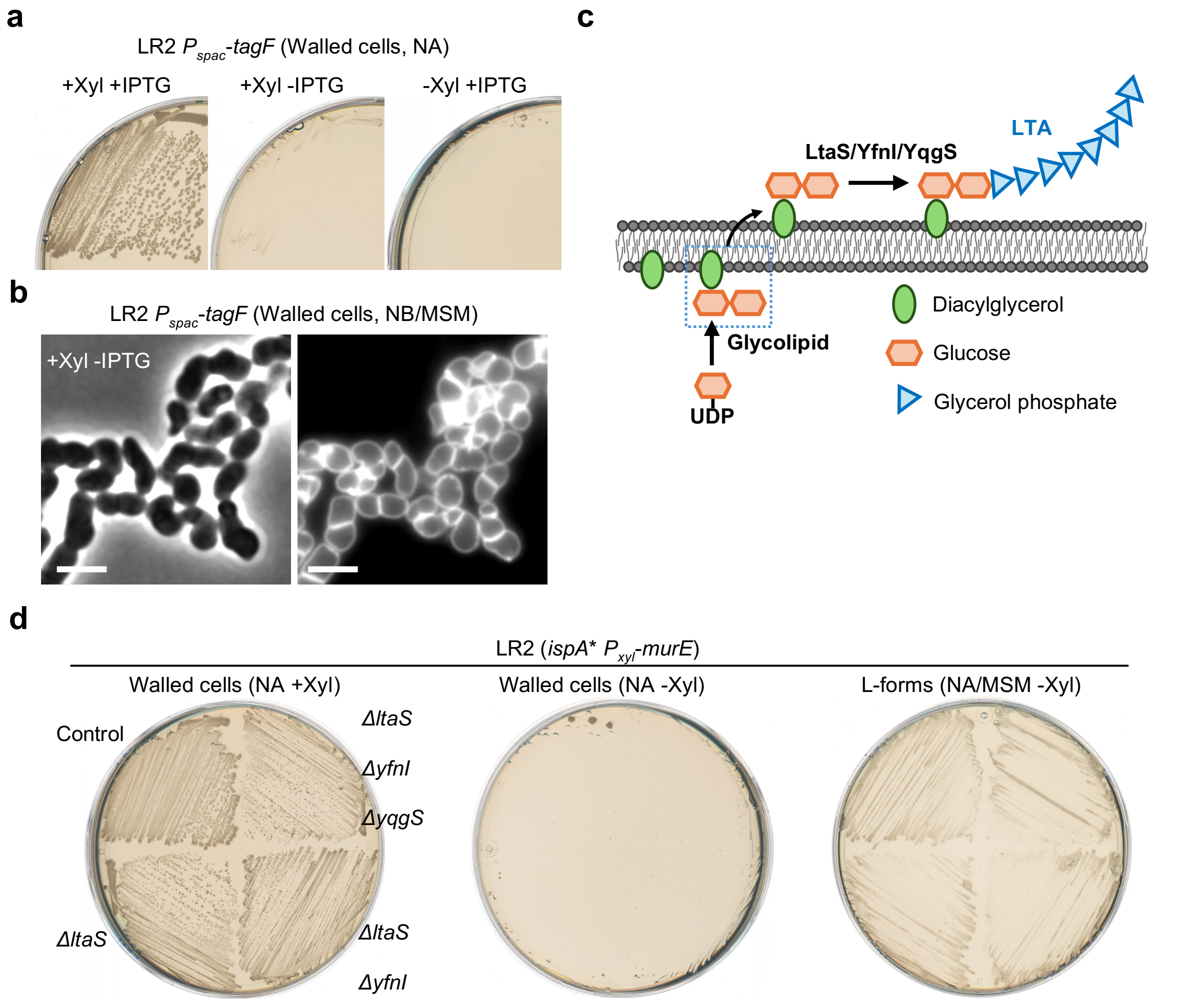

Supplementary Figure 3. The effects of *tagF* repression and LTA mutations on *B. subtilis* growth.
a. Growth of strains YK1361 (P_{spac}-*tagF ispA** P_{xyl}-*murE*) on NA plates with or without xylose (1 %) and IPTG (0.5 mM), as indicated.
b. A phase contrast micrograph and the corresponding Nile Red membrane staining image of YK1361 in NB/MSM containing xylose, but not IPTG. Scale bar represents 5 µm.
c. Schematic representation of LTA synthesis in *B. subtilis*.
d. L-form growth in the absence of LTA synthetic genes. LR2 (*ispA** P_{xyl}-*murE*), YK2390 (*ΔltaS ispA** P_{xyl}-*murE*), YK2392 (*ΔltaS ΔyfnI ispA** P_{xyl}-*murE*) and YK2394 (*ΔltaS ΔyfnI ΔyqgS ispA** P_{xyl}-*murE*) were streaked on NA plates, either with or without 0.5 % xylose, and NA/MSM plates without xylose, as indicated.

The experimental data in this figure are representative of multiple independent experiments.

Supplementary Table 1. Bacterial strains

Strains	Genotypes	References
168CA (wild-type)	*trpC2*	Lab. stock
BS115	*trpC2 cat-*P_{xyl}*-murE*	[1]
LR2	*trpC2 cat-*P_{xyl}*-murE* $ispA^{-}$	[1]
YK1334	*trpC2 ΔponA::spc*	[2]
YK1343	*trpC2 ΔrodA::neo*	[3]
YK1359	LR2 *ΩtagO::erm-*P_{spac}*-tagO*	[1,4]
YK1361	LR2 *ΩtagF::erm-*P_{spac}*-tagF*	[1,4]
YK1395	*trpC2* $ispA^{*}$ (*xseB::Tn-kan*)	[1]
YK1402	*trpC2 ΩtagO::erm-*P_{spac}*-tagO*	[4]
YK1405	*trpC2* $ispA^{*}$ (*xseB::Tn-kan*) *ΩtagO::erm-*P_{spac}*-tagO*	[1,4]
YK1464	LR2 *ΩtagO::erm-*P_{spac}*-tagO Δndh::Tn-kan*	[1,4,5]
YK1465	LR2 *ΩtagO::erm-*P_{spac}*-tagO ΔqoxB::Tn-kan*	[1,4,5]
YK1526	LR2 *ΩtagO::erm-*P_{spac}*-tagO ΔmhqR::Tn-kan*	[1,4,5]
YK1563	LR2 *ΩglmM::erm-*P_{spac}*-glmM-glmS*	[6]
YK2239	*trpC2 Δ4* (*ΔponA ΔpbpD ΔpbpF ΔpbpG*)	[7]
YK2390	LR2 *ΔltaS::erm*	[1,8]
YK2392	LR2 *ΔltaS::erm ΔyfnI::spc*	[1,8]
YK2394	LR2 *ΔltaS::erm ΔyfnI::spc ΔyfnI::kan*	[1,8]
YK2584	*trpC2 Δ4 ΩtagO::erm-*P_{spac}*-tagO*	[4,7]
YK2798	*trpC2* $ispA^{*}$ (*xseB::Tn-kan*) *ΔtagT::spc ΔtagU::cat ΩtagV::erm-*P_{spac}*-tagV*	[1,3]
YK2809	BS115 *ΩglmU::erm-*P_{spac}*-glmU*	[1,9]
YK2810	LR2 *ΩglmU::erm-*P_{spac}*-glmU*	[1,9]
YK2837	*trpC2 ΔrodA ΩtagO::erm-*P_{spac}*-tagO*	[3,4]
YK2911	LR2 *ΔltaS::neo ΔyfnI::spc ΩtagF::erm-*P_{spac}*-tagF*	[1,4,8]

Supplementary References

1 Mercier, R., Kawai, Y. & Errington, J. Excess membrane synthesis drives a primitive mode of cell proliferation. *Cell* **152**, 997-1007 (2013). https://doi.org:10.1016/j.cell.2013.01.043

2 Kawai, Y., Daniel, R. A. & Errington, J. Regulation of cell wall morphogenesis in *Bacillus subtilis* by recruitment of PBP1 to the MreB helix. *Mol Microbiol.* **71**, 1131-1144 (2009). https://doi.org:10.1111/j.1365-2958.2009.06601.x

3 Kawai, Y. *et al.* A widespread family of bacterial cell wall assembly proteins. *The EMBO journal* **30**, 4931-4941 (2011). https://doi.org:10.1038/emboj.2011.358

4 Yamamoto, H., Miyake, Y., Hisaoka, M., Kurosawa, S. & Sekiguchi, J. The major and minor wall teichoic acids prevent the sidewall localization of vegetative DL-endopeptidase LytF in *Bacillus subtilis*. *Mol Microbiol.* **70**, 297-310 (2008). https://doi.org:10.1111/j.1365-2958.2008.06397.x

5 Kawai, Y. *et al.* Cell growth of wall-free L-form bacteria is limited by oxidative damage. *Curr Biol* **25**, 1613-1618 (2015). https://doi.org:10.1016/j.cub.2015.04.031

6 Kawai, Y. *et al.* Crucial role for central carbon metabolism in the bacterial L-form switch and killing by β-lactam antibiotics. *Nature Microbiol.* (2019). https://doi.org:10.1038/s41564-019-0497-3

7 Emami, K. *et al.* RodA as the missing glycosyltransferase in *Bacillus subtilis* and antibiotic discovery for the peptidoglycan polymerase pathway. *Nat Microbiol.* **2**, 16253 (2017). https://doi.org:10.1038/nmicrobiol.2016.253

8 Schirner, K., Marles-Wright, J., Lewis, R. J. & Errington, J. Distinct and essential morphogenic functions for wall- and lipo-teichoic acids in *Bacillus subtilis*. *The EMBO journal* **28**, 830-842 (2009). https://doi.org:10.1038/emboj.2009.25

9 Kawai, Y. *et al.* On the mechanisms of lysis triggered by perturbations of bacterial cell wall biosynthesis. *Nat Commun.* **14**, 4123 (2023). https://doi.org:10.1038/s41467-023-39723-8
