# Supplementary material for: Synergistic action of peptidoglycan and teichoic acid synthesis inhibitors leads to cell death by oxidative damage
Source: Commun Biol. 2026 Apr 30;9:912. doi: 10.1038/s42003-026-10124-z (PMC13338364; doi:10.1038/s42003-026-10124-z)
Supplement: Supplementary file 2 — Description of Additional Supplementary Files [file 42003_2026_10124_MOESM2_ESM.docx]

**Description of Additional Supplementary File**

File name: Supplementary data
Description: The source data behind the graphs in the paper
