# Supplementary material for: Synergistic action of peptidoglycan and teichoic acid synthesis inhibitors leads to cell death by oxidative damage
Source: Commun Biol. 2026 Apr 30;9:912. doi: 10.1038/s42003-026-10124-z (PMC13338364; doi:10.1038/s42003-026-10124-z)
Supplement: Supplementary file 5 — Transparent Peer Review File [file 42003_2026_10124_MOESM5_ESM.pdf]

nature portfolio

Peer Review File

Synergistic action of peptidoglycan and teichoic acid synthesis inhibitors leads to cell death by oxidative damage

Corresponding Author: Dr Yoshikazu Kawai

This file contains all reviewer reports in order by version, followed by all author rebuttals in order by version.

Version 0:

Reviewer comments:

Reviewer #1

(Remarks to the Author)
Bacterial cells are usually surrounded by peptidoglycan, an important structural component required for cell shape maintenance and that withstand turgor pressure. However, under isotonic conditions bacteria can switch to cell wall-free state, the so-called L-forms, which are resistant to cell-wall-targeting antibiotics. The authors of the presented manuscript wanted to elucidate the requirement of wall and lipoteichoic acids, two important cell wall polymers, on the growth of L-forms. They could show that neither of the two is required for L-form growth. However, the enzyme responsible for the first committed step of WTA biosynthesis, TagO, is essential for L-form growth. The authors could elegantly show that the essentiality of TagO does not depend on WTA biosynthesis but rather the consumption on UDP-GlcNAc, which leads to reduced ROS stress. Lack of TagO, either by depletion or by tunicamycin inhibition, results in accumulation of UDP-GlcNAc and an increased metabolic flux leading to increased ROS-production. The increased levels of ROS ultimately cause the inability of L-forms to grow.
The authors used nice genetic approaches or antibiotics to test their hypotheses. The results are clearly presented and the conclusions are well supported by the obtained results.
The current work would benefit from an additional experiment, which would strengthen the results even further. As the authors describe, UDP-GlcNAc is synthesized by GlmS, GlmM and GlmU. This pathway can be inhibited by trans-cinnamaldehyde. If the inhibition of TagO only prevents L-form formation due to accumulation of UDP-GlcNAc, then L-forms should be able to grow in the presence of trans-cinnamaldehyde and tunicamycin (or in the tagO depletion strain).

While the study focusses on two Gram-positive bacteria, mainly Bacillus subtilis, the outcomes can likely be transferred to other Gram-positive bacteria and potentially also to Gram-negative bacteria as also outlined in the discussion of the paper. Furthermore, this paper clearly highlights the advantage of using L-forms to study cellular processes.
Minor comments:

Line 37: Insert “the” in front of “membrane”
Line 50: include the name of the ABC transporter
Lines 65/66: The authors state that β-lactam antibiotics block insertion of new lipid II, however, they only block TP activity and thus only prevent crosslinking of the glycan strands. Please rephrase
Line 131: Change “(bottom)” to “(Fig. 1e, bottom panel)”
Line 136: Correct “demonstrated”
Line 142: and 143: Change to “Fig. 2a, top” and “Fig. 2a, bottom”, respectively (and also for following Figures
Line 280: Remove the first “,”
Line 406: Change “caried” to “carried”
Line 413: Change “chlroramphenicol” to “chloramphenicol”
Fig. 4e: The strains of the first two plates are not indicated!

Reviewer #2

(Remarks to the Author)
This study investigates several aspects of the metabolic impact of the inhibition of cell-wall polymer synthesis in Bacillus subtilis. In a first set of experiments, the authors determined the essentiality of wall teichoic acid (WTA) and lipoteichoic acid

(LTA) in walled and L-form B. subtilis cells. This led to the surprising finding that TagO, which catalyzes the first committed step of WTA synthesis, is dispensable for growth of walled cells whereas it is essential for L-form cells. The essential role of TagO was not accounted for by the essentiality WTA because other enzymes in the teichoic acid synthesis pathway were dispensable in L-form cells. The basis for the essential role of TagO was explored by investigating the consequences of the reduced use of UDP-GlcNAc, the substrate of TagO, on other metabolic aspects. The absence of TagO was shown, as might have been expected, to result in an increase in the pool of UDP-GlcNAc. This is expected to lead to the feedback inhibition of GlmS, the enzyme that catalyzes the first step of UDP-GlcNAc synthesis from fructose-6 phosphate, and, in turn, to an increase in the metabolic flux in the glycolysis pathway. This led to the conclusion that a toxic effect results from the impaired use of UDP-GlcNAc following inhibition of either or both teichoic acid and peptidoglycan synthesis. The basis for this toxicity was further assigned to the formation of ROS. Together, the results provide evidence that an alteration of the central carbon oxidation pathway (glycolysis, TCA cycle and respiratory chain) rather than the lack of any specific cell wall polymer, was responsible for the inhibition of bacterial growth. These findings contribute to a better understanding of the mode of action of antibiotics that inhibit cell wall synthesis. These results also open new perspectives for the development of drug combinations that could act synergistically, as inferred from the synergy resulting from the inhibition of both peptidoglycan and teichoic acid synthesis.

From an experimental point of view, the originality of the study comes from the use of both walled and L-form cells as tractable models for bacteria producing peptidoglycan or not. The conclusions are justified as they are based on extensive investigations of both enzyme inhibition by drugs and down regulation of enzyme production using genetic constructs. There are no major critics related to the experimental design and the conclusions. The manuscript is concise and clearly written.

Major comment
1. The authors propose in the abstract and elsewhere in the text that UDP-GlcNAc controls a key metabolic switch leading either to balanced growth or oxidative damage. Although this statement is correct it is somewhat misleading because it could imply that bacterial cells respond to UDP-GlcNAc concentration via the binding of this nucleotide to proteins regulating gene expression. Instead, the data support the notion that a reduced use of fructose 6-phosphate in the UDP-MurNAc synthesis pathway results in an increase in the metabolic flux in the glycolysis pathway and consequently in the TCA cycle and the respiratory chain, the consequence of this catabolic burst being a stimulation of the generation of ROS as by-products. Thus, the growth of L-form cells is not dependent on the level of intracellular UDP-GlcNAc (Lines 363-364) but on the ability of the GlmS/U/V pathway to consume sufficient amounts of fructose-6-phosphate for preventing the catabolic burst leading to ROS production. The reader would benefit from a more detailed legend to Figure 7 summarizing the model and data supporting the model.

2. Fig. 6b and discussion. There is a ca. 40% increase in the amount of UDP-GlcNAc upon exposure of cells to LT-M. Would this be sufficient for feedback inhibition of GlmS? Is the Ki known for the B. subtilis enzyme? The moderate accumulation of UDP-GlcNAc may imply that this precursor is consumed in other pathways. The authors should consider presenting these alternative possibilities in more details.

Minor points

Lines 39 and 40. "… requires the insertion of a PG precursor molecule, called lipid II." The statement is not precise: Lipid II, as such, is not inserted into the wall. It is the disaccharide-peptide moiety of lipid II which is inserted.

Line 50. "by an ABC transporter"

Lines 59 and 60. Please indicate the function of LtaS. The authors should consider introducing a figure describing the LTA assembly pathway, as this is investigated in the current study

Line 116. …lipid II synthesis to be shut down in the absence of xylose.

Line 121. (Figs.2a, b); missing space.

Line 142. (Fig 2a,) add missing letter or delete comma.

Line 231. The signals should be defined.

Line 265. Fosfomycin without capital F.

Line 326. certain

Line 419. "OD units 60 = OD600 x 100 µl of the culture" The actual cell density in the disk diffusion assay should be specified.

Michel Arthur

Reviewer #3

(Remarks to the Author)

In this manuscript, Kawai et al. identify that the intracellular concentration of UDP-GlcNAc — the precursor for both PG and WTA — is important for L-form growth. Their work convincingly shows that during normal L-from growth, WTA continues to be synthesized, keeping UDP-GlcNAc levels below a threshold that triggers feedback inhibition of GlmS, thereby enabling the synthesis of more UDP-GlcNAc for WTA synthesis, maintaining glycolytic homeostasis and limiting the production of reactive oxygen species. Inhibition of WTA synthesis leads to accumulation of unused UDP-GlcNAc, GlmS inhibition, and increased flux into the glycolytic pathway, which increases ROS and oxidative damage.

This is a thorough and compelling study that links the metabolic homeostasis of cell wall precursors to central carbon metabolism. It demonstrates how disruption of this coupling leads to oxidative killing and helps explain the mode of action of cell wall–active antibiotics. The work also provides new insight into the role of WTAs in the MreB–Rod system, showing that WTAs somehow guide MreB–RodA activity. This finding adds nuance to earlier observations from Suzanne Walker's group, which showed that inhibition of late steps in WTA synthesis—through sequestration of C55 in WTA precursors—disrupts MreB filament formation. Although the precise mechanism by which WTAs influence MreB–RodA was not resolved, this does not detract from the central conclusion. Instead, the study underscores the multifaceted role of WTAs in peptidoglycan biosynthesis and highlights their importance in limiting oxidative stress in L-forms.

Overall, this work is exceptional, and I have no major concerns. There were only two minor points below:

Minor points:

Figure 1: "Lys" is used to abbreviate both lysostaphin and lysozyme, which is somewhat confusing. As these two enzymes have different PG and species specificities. I suggest using different abbreviations.

Line 184: Please clarify how the different phenotypes (bulged v.s. spherical) arise. Is it due to differential inhibition of late v.s. early biosynthetic steps?

Version 1:

Reviewer comments:

Reviewer #1

(Remarks to the Author)
I am happy with the changes! Great work.

Reviewer #2

(Remarks to the Author)
All comments have been taken into account and I have no further suggestions for improvment.

Reviewer #3

(Remarks to the Author)
All of my points were addressed, and I have no further comments. Congratulations on this great piece of work!

Open Access This Peer Review File is licensed under a Creative Commons Attribution 4.0 International License, which permits use, sharing, adaptation, distribution and reproduction in any medium or format, as long as you give appropriate credit to the original author(s) and the source, provide a link to the Creative Commons license, and indicate if changes were made.
In cases where reviewers are anonymous, credit should be given to 'Anonymous Referee' and the source.
The images or other third party material in this Peer Review File are included in the article's Creative Commons license, unless indicated otherwise in a credit line to the material. If material is not included in the article's Creative Commons license and your intended use is not permitted by statutory regulation or exceeds the permitted use, you will need to obtain permission directly from the copyright holder.
To view a copy of this license, visit https://creativecommons.org/licenses/by/4.0/

Your manuscript entitled "Synergistic action of peptidoglycan and teichoic acid synthesis inhibitors leads to cell death by oxidative damage" has now been seen by 3 referees, whose comments are appended below. You will see from their comments below that while they find your work of considerable interest, some important points are raised. We are interested in the possibility of publishing your study in Communications Biology, but would like to consider your response to these concerns in the form of a revised manuscript before we make a final decision on publication.
>>> Thanks for your handling our paper. We were pleased to see positive remarks from all the reviewers. We provide a point-by-point response to all of the reviewers' comments and questions, along with the revised manuscript. We have also amended the manuscript in accordance with the formatting guidelines. All changes to the text have been highlighted in yellow.

We therefore invite you to revise and resubmit your manuscript, taking into account the points raised. In particular, assessing the effect of trans-cinnamaldehyde, which specifically inhibits TagO, would provide additional evidence to strengthen the conclusions drawn further.
>>> Trans-cinnamaldehyde (tCA) is a natural antibacterial agent that is effective against a broad spectrum of Gram-positive and Gram-negative bacteria, as well as fungi and yeasts (e.g. the review paper by Jimenez, 2024; doi: 10.3390/antibiotics13111095). It appears to act primarily by disrupting cell membranes, causing cytoplasmic leakage and a reduction in intracellular ATP levels. Interestingly, tCA also interferes with the UDP-GlcNAc homeostasis in *Listeria monocytogenes* likely by limiting the availability of the substrate fructose-6P (Sun 2021; doi: 10.3390/foods10071666), as noted by Reviewer 1. However, the primary targets of tCA remain unclear (see also below).

In this paper, we used tunicamycin at the low concentrations (below 5 µg/ml), which are commonly used to specifically inhibit TagO and WTA synthesis in extensive studies of *B. subtilis* and *S. aureus*. We also confirmed the effect of TagO inhibition by genetically repressing and restoring *tagO* gene expression, using an IPTG-dependent promoter. We have done some preliminary experiments to look at tCA (see comments below) without getting clear cut results. As the reviewer did not seem to have strong feelings about this we propose to leave a more detailed analysis for a future paper.

Reviewers' comments:

Reviewer #1 (Remarks to the Author):

Bacterial cells are usually surrounded by peptidoglycan, an important structural component required for cell shape maintenance and that withstand turgor pressure. However, under isotonic conditions bacteria can switch to cell wall-free state, the so-

called L-forms, which are resistant to cell-wall-targeting antibiotics. The authors of the presented manuscript wanted to elucidate the requirement of wall and lipoteichoic acids, two important cell wall polymers, on the growth of L-forms. They could show that neither of the two is required for L-form growth. However, the enzyme responsible for the first committed step of WTA biosynthesis, TagO, is essential for L-form growth. The authors could elegantly show that the essentiality of TagO does not depend on WTA biosynthesis but rather the consumption on UDP-GlcNAc, which leads to reduced ROS stress. Lack of TagO, either by depletion or by tunicamycin inhibition, results in accumulation of UDP-GlcNAc and an increased metabolic flux leading to increased ROS-production. The increased levels of ROS ultimately cause the inability of L-forms to grow.
The authors used nice genetic approaches or antibiotics to test their hypotheses. The results are clearly presented and the conclusions are well supported by the obtained results.
The current work would benefit from an additional experiment, which would strengthen the results even further. As the authors describe, UDP-GlcNAc is synthesized by GlmS, GlmM and GlmU. This pathway can be inhibited by trans-cinnamaldehyde. If the inhibition of TagO only prevents L-form formation due to accumulation of UDP-GlcNAc, then L-forms should be able to grow in the presence of trans-cinnamaldehyde and tunicamycin (or in the tagO depletion strain).
>>> This is an interesting suggestion. We have done some preliminary experiments testing the effects of trans-cinnamaldehyde on L-form growth, but no informative results have emerged yet.

It has been suggested that this compound interferes with the UDP-GlcNAc homeostasis in *L. monocytogenes* lacking the YvcK (GlmR) protein, probably by reducing fructose-6P availability. Therefore, the results may also be influenced by YvcK and growth conditions (i.e. carbon source) in *B. subtilis* and L-forms. We plan to investigate this in the future but it would seem to take us beyond the scope of the current paper.

While the study focusses on two Gram-positive bacteria, mainly Bacillus subtilis, the outcomes can likely be transferred to other Gram-positive bacteria and potentially also to Gram-negative bacteria as also outlined in the discussion of the paper. Furthermore, this paper clearly highlights the advantage of using L-forms to study cellular processes.
>>> We greatly appreciate these positive comments. We have corrected all of the minor errors and omissions in the revised manuscript.

Minor comments:
Line 37: Insert “the” in front of “membrane”
Line 50: include the name of the ABC transporter
Lines 65/66: The authors state that β-lactam antibiotics block insertion of new lipid II, however, they only block TP activity and thus only prevent crosslinking of the glycan strands. Please rephrase
Line 131: Change “(bottom)” to “(Fig. 1e, bottom panel)”
Line 136: Correct “demonstrated”
Line 142: and 143: Change to “Fig. 2a, top” and “Fig. 2a, bottom”, respectively (and also for following Figures

Line 280: Remove the first “,”
Line 406: Change “caried” to “carried”
Line 413: Change “chlroramphenicol” to “chloramphenicol”
Fig. 4e: The strains of the first two plates are not indicated!
>>> All corrected.

Reviewer #2 (Remarks to the Author):

This study investigates several aspects of the metabolic impact of the inhibition of cell-wall polymer synthesis in Bacillus subtilis. In a first set of experiments, the authors determined the essentiality of wall teichoic acid (WTA) and lipoteichoic acid (LTA) in walled and L-form B. subtilis cells. This led to the surprising finding that TagO, which catalyzes the first committed step of WTA synthesis, is dispensable for growth of walled cells whereas it is essential for L-form cells. The essential role of TagO was not accounted for by the essentiality WTA because other enzymes in the teichoic acid synthesis pathway were dispensable in L-form cells. The basis for the essential role of TagO was explored by investigating the consequences of the reduced use of UDP-GlcNAc, the substrate of TagO, on other metabolic aspects. The absence of TagO was shown, as might have been expected, to result in an increase in the pool of UDP-GlcNAc. This is expected to lead to the feedback inhibition of GlmS, the enzyme that catalyzes the first step of UDP-GlcNAc synthesis from fructose-6 phosphate, and, in turn, to an increase in the metabolic flux in the glycolysis pathway. This led to the conclusion that a toxic effect results from the impaired use of UDP-GlcNAc following inhibition of either or both teichoic acid and peptidoglycan synthesis. The basis for this toxicity was further assigned to the formation of ROS. Together, the results provide evidence that an alteration of the central carbon oxidation pathway (glycolysis, TCA cycle and respiratory chain) rather than the lack of any specific cell wall polymer, was responsible for the inhibition of bacterial growth. These findings contribute to a better understanding of the mode of action of antibiotics that inhibit cell wall synthesis. These results also open new perspectives for the development of drug combinations that could act synergistically, as inferred from the synergy resulting from the inhibition of both peptidoglycan and teichoic acid synthesis.

From an experimental point of view, the originality of the study comes from the use of both walled and L-form cells as tractable models for bacteria producing peptidoglycan or not. The conclusions are justified as they are based on extensive investigations of both enzyme inhibition by drugs and down regulation of enzyme production using genetic constructs. There are no major critics related to the experimental design and the conclusions. The manuscript is concise and clearly written.
>>> We again greatly appreciate the valuable feedback and positive comments.

Major comment
1. The authors propose in the abstract and elsewhere in the text that UDP-GlcNAc controls a key metabolic switch leading either to balanced growth or oxidative damage. Although this statement is correct it is somewhat misleading because it could imply that bacterial cells respond to UDP-GlcNAc concentration via the binding of this nucleotide to proteins regulating gene expression. Instead, the data support the notion that a reduced use of fructose 6-phosphate in the UDP-MurNAc synthesis

pathway results in an increase in the metabolic flux in the glycolysis pathway and consequently in the TCA cycle and the respiratory chain, the consequence of this catabolic burst being a stimulation of the generation of ROS as by-products. Thus, the growth of L-form cells is not dependent on the level of intracellular UDP-GlcNAc (Lines 363-364) but on the ability of the GlmS/U/V pathway to consume sufficient amounts of fructose-6-phosphate for preventing the catabolic burst leading to ROS production. The reader would benefit from a more detailed legend to Figure 7 summarizing the model and data supporting the model.
>>> We have amended the discussion section (lines 364-380 in the revised manuscript) and the new legend for Figure 7 accordingly.

2. Fig. 6b and discussion. There is a ca. 40% increase in the amount of UDP-GlcNAc upon exposure of cells to LT-M. Would this be sufficient for feedback inhibition of GlmS? Is the Ki known for the B. subtilis enzyme? The moderate accumulation of UDP-GlcNAc may imply that this precursor is consumed in other pathways. The authors should consider presenting these alternative possibilities in more details.
>>> In. *B. subtilis*, it has been proposed that, at high concentration, UDP-GlcNAc acts as a feedback inhibitor of GlmS by antagonising its activator, GlmR. UDP-GlcNAc binds to GlmR, causing dissociation from GlmS and thereby preventing GlmS activity. A previous in vitro study showed that, at concentrations above 0.1 mM, UDP-GlcNAc can interfere with GlmS stimulation by GlmR (Foulquier et al., 2020, https://doi.org/10.1038/s41598-020-72854-2). We observed that the L-form cells began to lyse within 60 minutes when L-TM treatment commenced (Fig. 6b), but a longer treatment period might result in an even greater accumulation of UDP-GlcNAc. Nevertheless, we do not exclude the possibility that UDP-GlcNAc is also consumed in other pathways. We have added text to discuss this as requested.

Minor points

Lines 39 and 40. "… requires the insertion of a PG precursor molecule, called lipid II." The statement is not precise: Lipid II, as such, is not inserted into the wall. It is the disaccharide-peptide moiety of lipid II which is inserted.

>>> Corrected.

Line 50. "by an ABC transporter"
>>> Corrected.

Lines 59 and 60. Please indicate the function of LtaS. The authors should consider introducing a figure describing the LTA assembly pathway, as this is investigated in the current study
>>> The LtaS protein and its homologues polymerise the glycerol phosphate chain and link it to the glycolipid anchor, which is produced from UDP-glucose and diacylglycerol. We have added this to the Introduction section, and a schematic representation of LTA synthesis is now shown in new Supplementary Figure 3c.

Line 116. …lipid II synthesis to be shut down in the absence of xylose.
Line 121. (Figs.2a, b); missing space.
Line 142. (Fig 2a,) add missing letter or delete comma.
Line 231. The signals should be defined.

Line 265. Fosfomycin without capital F.
Line 326. Certain
Line 419. "OD units 60 = OD600 x 100 µl of the culture" The actual cell density in the disk diffusion assay should be specified.
>>> All corrected.

Michel Arthur

Reviewer #3 (Remarks to the Author):

In this manuscript, Kawai et al. identify that the intracellular concentration of UDP-GlcNAc — the precursor for both PG and WTA — is important for L-form growth. Their work convincingly shows that during normal L-from growth, WTA continues to be synthesized, keeping UDP-GlcNAc levels below a threshold that triggers feedback inhibition of GlmS, thereby enabling the synthesis of more UDP-GlcNAc for WTA synthesis, maintaining glycolytic homeostasis and limiting the production of reactive oxygen species. Inhibition of WTA synthesis leads to accumulation of unused UDP-GlcNAc, GlmS inhibition, and increased flux into the glycolytic pathway, which increases ROS and oxidative damage.

This is a thorough and compelling study that links the metabolic homeostasis of cell wall precursors to central carbon metabolism. It demonstrates how disruption of this coupling leads to oxidative killing and helps explain the mode of action of cell wall–active antibiotics. The work also provides new insight into the role of WTAs in the MreB–Rod system, showing that WTAs somehow guide MreB–RodA activity. This finding adds nuance to earlier observations from Suzanne Walker's group, which showed that inhibition of late steps in WTA synthesis—through sequestration of C55 in WTA precursors—disrupts MreB filament formation. Although the precise mechanism by which WTAs influence MreB–RodA was not resolved, this does not detract from the central conclusion. Instead, the study underscores the multifaceted role of WTAs in peptidoglycan biosynthesis and highlights their importance in limiting oxidative stress in L-forms.

Overall, this work is exceptional, and I have no major concerns. There were only two minor points below:
>>> We again greatly appreciate the positive comments.

Minor points:

Figure 1: "Lys" is used to abbreviate both lysostaphin and lysozyme, which is somewhat confusing. As these two enzymes have different PG and species specificities. I suggest using different abbreviations.
>>> Reworded as suggested. "Lys' for lysozyme and "Lys-s" for lysostaphin.

Line 184: Please clarify how the different phenotypes (bulged v.s. spherical) arise. Is it due to differential inhibition of late v.s. early biosynthetic steps?
>>> Downregulation of most biosynthetic genes in the WTA pathway (including both early and late genes) results in a similar morphological defect in *B. subtilis*. In

general, the cells initially swell and bulge, and then gradually transform into a spherical-like shape. However, it appears to be influenced by the growth conditions, such as nutrient availability and osmolarity.
